# Peptidoglycan Recognition Proteins Kill Bacteria by Inducing Oxidative, Thiol, and Metal Stress

**DOI:** 10.1371/journal.ppat.1004280

**Published:** 2014-07-17

**Authors:** Des Raj Kashyap, Annemarie Rompca, Ahmed Gaballa, John D. Helmann, Jefferson Chan, Christopher J. Chang, Iztok Hozo, Dipika Gupta, Roman Dziarski

**Affiliations:** 1 Indiana University School of Medicine–Northwest, Gary, Indiana, United States of America; 2 Department of Microbiology, Cornell University, Ithaca, New York, United States of America; 3 Departments of Chemistry and Molecular and Cell Biology and the Howard Hughes Medical Institute, University of California, Berkeley, Berkeley, California, United States of America; 4 Department of Mathematics, Indiana University Northwest, Gary, Indiana, United States of America; University of Toronto, Canada

## Abstract

Mammalian Peptidoglycan Recognition Proteins (PGRPs) are a family of evolutionary conserved bactericidal innate immunity proteins, but the mechanism through which they kill bacteria is unclear. We previously proposed that PGRPs are bactericidal due to induction of reactive oxygen species (ROS), a mechanism of killing that was also postulated, and later refuted, for several bactericidal antibiotics. Here, using whole genome expression arrays, qRT-PCR, and biochemical tests we show that in both *Escherichia coli* and *Bacillus subtilis* PGRPs induce a transcriptomic signature characteristic of oxidative stress, as well as correlated biochemical changes. However, induction of ROS was required, but not sufficient for PGRP killing. PGRPs also induced depletion of intracellular thiols and increased cytosolic concentrations of zinc and copper, as evidenced by transcriptome changes and supported by direct measurements. Depletion of thiols and elevated concentrations of metals were also required, but by themselves not sufficient, for bacterial killing. Chemical treatment studies demonstrated that efficient bacterial killing can be recapitulated only by the simultaneous addition of agents leading to production of ROS, depletion of thiols, and elevation of intracellular metal concentrations. These results identify a novel mechanism of bacterial killing by innate immunity proteins, which depends on synergistic effect of oxidative, thiol, and metal stress and differs from bacterial killing by antibiotics. These results offer potential targets for developing new antibacterial agents that would kill antibiotic-resistant bacteria.

## Author Summary

Bacterial infections are still a major cause of morbidity and mortality because of increasing antibiotic resistance. New targets for developing new approaches to antibacterial therapy are needed, because discovering new or improving current antibiotics have become increasingly difficult. One such approach is developing new antibacterial agents based on the antibacterial mechanisms of bactericidal innate immunity proteins, such as human peptidoglycan recognition proteins (PGRPs). Thus, our aim was to determine how PGRPs kill bacteria. We previously proposed that PGRPs kill bacteria by inducing toxic oxygen by-products (“reactive oxygen species”, ROS) in bacteria. It was also previously proposed, but recently refuted, that bactericidal antibiotics kill bacteria by inducing ROS production in bacteria. These findings prompted us to evaluate in greater detail the mechanism of PGRP-induced bacterial killing, including the role of ROS in PGRP killing. We show here that PGRPs kill bacteria through synergistic induction of ROS, depletion of thiols, and increasing intracellular concentration of metals, which are all required, but individually not sufficient for bacterial killing. Our results reveal a novel bactericidal mechanism of innate immunity proteins, which differs from killing by antibiotics and offers alternative targets for developing new antibacterial therapies for antibiotic-resistant bacteria.

## Introduction

Mammalian Peptidoglycan Recognition Proteins (PGRPs) are a family of four evolutionary conserved antibacterial innate immunity proteins [1]–[3]. Three PGRPs (PGLYRP1, PGLYRP3, and PGLYRP4) are directly bactericidal [4], [5] and one PGRP (PGLYRP2) is a peptidoglycan-lytic amidase [6]. PGRPs kill both Gram-positive and Gram-negative bacteria [4], [5] by a novel mechanism [7]. PGRPs activate envelope stress responses in bacteria, which results in membrane depolarization and intracellular production of toxic hydroxyl radicals (HO^•^), which leads to energy depletion and inhibition of intracellular synthesis of peptidoglycan, proteins, RNA, and DNA, and cell death [7]. Bactericidal PGRPs do not inhibit extracellular peptidoglycan synthesis, do not hydrolyze the cell wall, and do not kill by permeabilizing bacterial membranes, or by osmotic lysis [4], [5], [7]. The induction of envelope stress by PGRPs in two model Gram-positive and Gram-negative bacteria is to a large extent dependent on the inappropriate over-activation of two-component systems that normally function to detect and dispose of misfolded proteins in bacteria, CssRS in *Bacillus subtilis*, and CpxRA in *Escherichia coli* [7]. The exact nature of the signal that activates CssRS and CpxRA is not known, because these two-component systems respond to many other types of stress besides misfolded proteins, including pH, osmolarity, Cu, and Zn [8].

In this study we investigate the down-stream events that are responsible for PGRP-induced bacterial killing. We first focused on the role of oxidative stress and reactive oxygen species (ROS) in PGRP bacterial killing, because we could inhibit bacterial killing by inhibiting PGRP-induced HO^•^ production [7]. Detailed evaluation of the role of ROS in PGRP-induced killing was important, because the previously reported antibiotic-induced killing of *E. coli* that was also based on CpxRA-dependent induction of HO^•^ [9], [10] was called into question by recent reports showing that antibiotic-mediated killing of *E. coli* does not depend on ROS, as bactericidal antibiotics did not induce H_2_O_2_ production or corresponding oxidative stress responses that would signal the presence of elevated levels of H_2_O_2_ [11]–[13].

Our results presented here show remarkably similar responses to PGRPs in both *E. coli* and *B. subtilis*. Both model organisms displayed similar transcriptomic signatures upon treatment with PGRPs, including induction of oxidative, thiol, and metal stress responses, along with corresponding increases in intracellular H_2_O_2_ and metals and depletion of thiols. We demonstrate that all these three responses are required, but individually are not sufficient for bacterial killing by PGRPs. We further show that bacterial killing can be efficiently reconstituted by the simultaneous treatment with chemicals that lead to production of ROS, depletion of thiols, and elevation of intracellular metal concentrations. These results indicate that killing of bacteria by PGRPs involves synergistic effects of oxidative, thiol, and metal stress and is different than killing by antibiotics.

## Results

### PGRPs induce oxidative, thiol, and metal stress genes in bacteria

To gain further insights into the mechanism(s) of PGRP-mediated killing of bacteria, we used the unbiased approach of whole genome expression arrays to identify stress response pathways activated in PGRP-treated bacteria. We treated bacteria with human PGRP and after 30 min we isolated RNA (before the numbers of viable bacteria recovered by colony counts began to significantly decrease). We used albumin as a negative control, and we used two well-characterized bactericidal compounds as controls. The first was gentamicin, an antibiotic that activates the same misfolded protein-sensing two-component systems as PGRP [7], [10], but which was also recently shown *not* to induce H_2_O_2_ production or oxidative stress responses in *E. coli* [12]. The second was CCCP (carbonyl cyanide 3-chlorophenylhydrazone), a membrane potential de-coupler, which, similar to PGRP, induces membrane depolarization in bacteria [7].

Using whole genome expression arrays in three independent experiments we detected expression of 5,531 probes in *E. coli* and 3,355 probes in *B. subtilis*, of which 1,510 and 536 probes were expressed significantly higher in PGRP-treated *E. coli* and *B. subtilis*, respectively, than in albumin-treated bacteria, and 1,988 and 617 probes were expressed significantly lower in PGRP-treated *E. coli* and *B. subtilis*, respectively, than in albumin-treated bacteria (as determined by one-tailed *t*-test at *P*≤0.05). Further calculation of FDR (false discovery rate) *q* values identified 2,733 and 795 probes in *E. coli* and *B. subtilis*, respectively, whose expression was significantly changed in PGRP-treated compared with albumin-treated bacteria at *q*≤0.05. In *E. coli* 2,008 genes and in *B. subtilis* 1,236 genes were either up-regulated or down-regulated more than 3 times by any of the three treatments (PGRP, gentamicin, and CCCP, Figures S1 and S2). We confirmed increased expression of representative 25 *E. coli* and 28 *B. subtilis* up-regulated genes using quantitative real time PCR (qRT-PCR, Tables S1 and S2).

**Figure 1:**
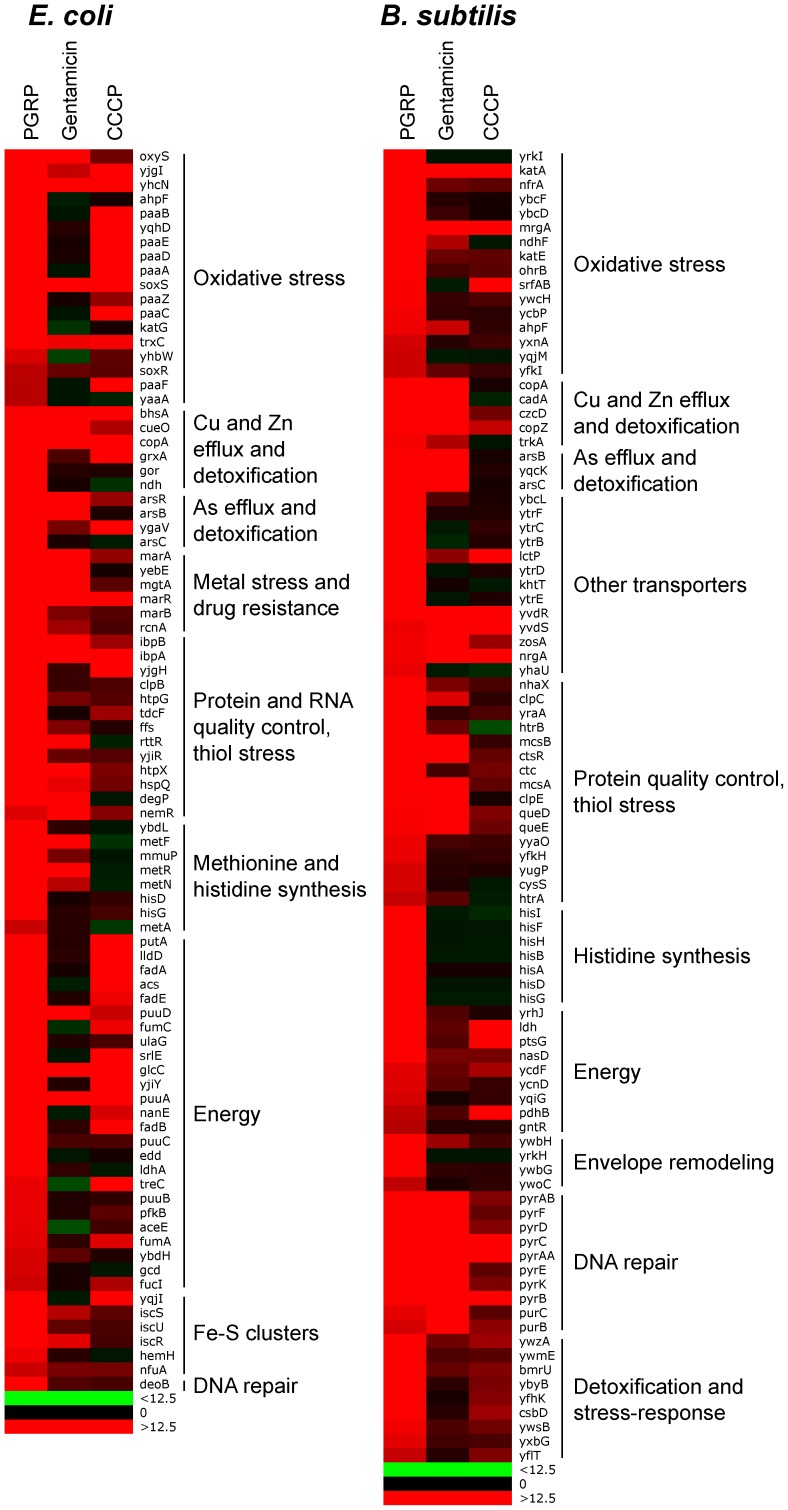
Top up-regulated groups of genes in PGRP-treated *E. coli* and *B. subtilis*. Bacteria were treated with PGRP (PGLYRP4), gentamicin, or CCCP as in Tables 1, S1 and S2 and the fold increase in gene expression was determined by whole genome expression arrays. The results are means of 3 experiments and the quantitative expression data, the significance of differences, and the gene functions and regulators are shown in Tables S1 and S2.

The results showed remarkably similar effects of PGRP on gene expression in *E. coli* and *B. subtilis*. Virtually all top PGRP-induced genes were involved in defense against oxidative, thiol (disulfide), and metal stress, or in repair of the cellular damage in bacteria caused by these stresses (Figure 1 and Tables 1 and S1, S2, S3, S4). They included: (i) peroxide detoxification genes (*oxyS*, *ahpF*, *katG* in *E. coli*, and *katA*, *katE*, *ohrB*, *ahpF*, *ahpC* in *B. subtilis*) induced by peroxide-responsive OxyR in *E. coli*, and PerR in *B. subtilis*; (ii) genes involved in detoxification of ROS and epoxides (*paa* operon in *E. coli* controlled by Crp and Ihf); (iii) genes involved in efflux and detoxification of copper, zinc, arsenite, and other metals induced by metal-responsive or stress-responsive regulators (CueR, ArsR, SoxR, RcnR in *E. coli*, and CsoR, CzrA, ArsR in *B. subtilis*); (iv) genes coding for chaperones and protein, RNA, and DNA quality control induced by stress-responsive regulators (σ^H^, Ihf, CpxRA in *E. coli*, and σ^B^, CtsR, CssRS in *B. subtilis*); and (v) genes for repair and synthesis of Fe-S clusters (controlled by IscR in *E. coli*). The remaining groups of highly induced genes also reflect bacterial response to oxidative, thiol, and metal stress and function in energy generation, synthesis or uptake of methionine and histidine, and defense against general stress (Figure 1 and Tables 1, S1 and S2).

**Table 1:** Top up-regulated and down-regulated processes and regulons/operons in PGRP-treated *E. coli* and *B. subtilis* a.

Processes	Regulons/Operons	
**Up-regulated**	***E. coli***	***B. subtilis***
Oxidative and thiol stress response	OxyR, Crp, Ihf, SoxR, Fnr	σ^B^, PerR
Metal efflux and detoxification	CueR, ArsR, SoxR, RcnR	CsoR, CzrA, ArsR
Chaperones, protein and RNA quality control	σ^H^, Ihf, CpxRA	σ^B^, CtsR, CssRS
Carbohydrate and fatty acid metabolism	*fad, fuc, fum* operons	N/A
Amino acid biosynthesis	*met*, *his* operons	*his* operon
Nucleotide biosynthesis	*deo* operon	*pyr, pur* operons
Fe-S cluster biosynthesis	IscR	N/A
**Down-regulated**	***E. coli***	***B. subtilis***
Iron uptake	Fur	Fur
Motility	CpxRA, FlhDC	σ^D^
Phosphate utilization and uptake	N/A	PhoPR

a Bacteria were treated with PGRP (PGLYRP4) for 30 min at 37°C and gene expression was determined by whole genome expression arrays and qRT-PCR. The mean quantitative expression data for individual genes, the significance of differences, and gene functions and regulators are shown in Tables S1, S2, S3, S4. Data for the entire whole genome expression arrays have been deposited in NCBI GEO under the accession numbers GSE44211 and GSE44212.

The majority of genes highly up-regulated by PGRP were not induced or induced less by gentamicin and CCCP (Figures 1, S1 and S2, Tables S1 and S2). Many oxidative stress, energy acquisition, and methionine and histidine biosynthesis genes (in both *E. coli* and *B. subtilis*), and some metal detoxification and Fe-S biosynthesis genes (in *E. coli*) and genes for transporters, envelope remodeling, and general stress response (in *B. subtilis*) were induced less (or not at all) by gentamicin compared with PGRP. However, both PGRP and gentamicin induced SoxR-regulated *soxS* and *marRAB* genes (which control drug resistance in *E. coli*) and several genes for protein quality control (in both *E. coli* and *B. subtilis*). The gene induction patterns by CCCP in *E. coli* and *B. subtilis* were also unique and different from the pattern induced by PGRP or gentamicin, with induction of several oxidative stress genes and energy acquisition genes, and some metal detoxification genes (Figure 1 and Table S1).

Different patterns of gene activation by PGRP and other antibacterial compounds and also overlapping activation of genes by PGRP for oxidative, thiol, metal, and also envelope stress were further revealed by hierarchical cluster analysis by comparing PGRP-activated genes with previously published gene array data in bacteria exposed to H_2_O_2_, diamide (thiol-oxidizing agent), Zn, and vancomycin (inhibitor of peptidoglycan synthesis). This analysis revealed clusters of genes induced primarily by PGRP (e.g., several OxyR-induced and DNA repair genes), and several clusters of PGRP-induced genes overlapping with genes induced either by H_2_O_2_, or diamide, or Zn, or vancomycin (Figure S3). Altogether, our gene expression results suggest simultaneous induction of multiple stress responses by PGRP.

Inspection of genes down-regulated after PGRP treatment was also informative. The most down-regulated genes in both *E. coli* and *B. subtilis* were for: (i) Fe uptake, controlled by the Fur regulator in both bacteria; (ii) motility, controlled by CpxRA in *E. coli*; and (iii) phosphate utilization, controlled by PhoPR in *B. subtilis* (Figures S1 and S2, Tables 1, S3 and S4).

Thus, our gene expression results indicate that PGRPs induce oxidative stress, thiol stress, and metal stress in bacteria, and our next experiments were designed to verify these responses biochemically and to determine which of these responses are involved in bacterial killing.

### PGRPs induce production of H_2_O_2_

In both *E. coli* and *B. subtilis*, PGRPs induced expression of genes typical of oxidative stress, including genes regulated by intracellular peroxide sensors, OxyR and PerR (Tables 1, S1 and S2). We therefore tested the hypothesis that PGRPs induce production of H_2_O_2_ in bacteria. Oxidative stress can arise from the intracellular production of superoxide anion (O_2_^−^), which is then converted into hydrogen peroxide (H_2_O_2_) and then into HO^•^, which are collectively known as ROS [14]. Thus, our hypothesis was also consistent with our previous results showing induction of HO^•^ by PGRPs in bacteria [7]. To directly verify this hypothesis, we measured production of H_2_O_2_, because H_2_O_2_ is more stable than other ROS (O_2_^−^ and HO^•^) and diffuses readily across membranes facilitating its detection. To detect H_2_O_2_ production, we used mutants, designated Hpx^−^, deficient in the major H_2_O_2_ degrading enzymes catalase (*kat*) and alkyl hydroperoxide reductase (*ahp*) (*E. coli* Δ*katG*Δ*katE*Δ*ahpCF* and *B. subtilis* Δ*katA*Δ*ahpCF*) [12], [15]–[17].

**Figure 2:**
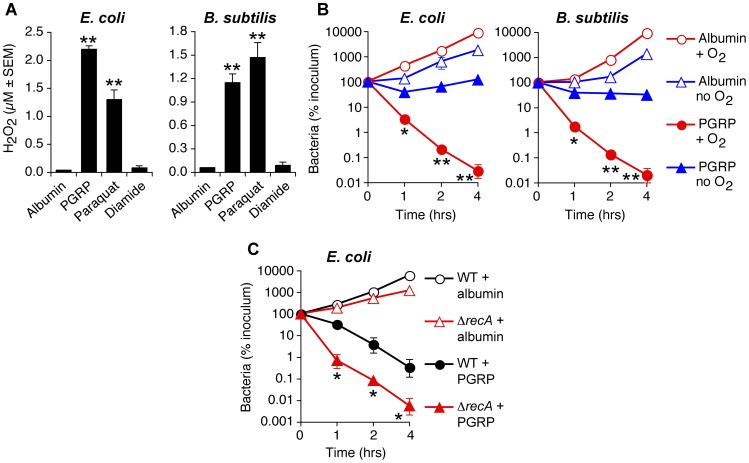
PGRP induces H_2_O_2_ production, requires O_2_ for killing, and the killing involves DNA damage. (**A**) Hpx^−^ *E. coli* or *B. subtilis* were incubated aerobically with albumin (50 µg/ml), PGRP (PGLYRP3, 50 µg/ml), paraquat (5 µM), or diamide (250 µM) for 15 min and H_2_O_2_ production was measured. (**B**) *E. coli* or *B. subtilis* were incubated aerobically (+O_2_) or anaerobically (*E. coli*) or under microaerophilic conditions (*B. subtilis*) (no O_2_) with 50 µg/ml PGRP (PGLYRP4) or albumin and the numbers of bacteria were determined. (**C**) WT or Δ*recA E. coli* were incubated aerobically with 30 µg/ml PGRP (PGLYRP3) or albumin and the numbers of bacteria were determined. The results are means ± SEM of 3 experiments (SEM were within symbols, if not visible); each experiment was repeated once with rMSA and another PGRP with similar results (A and B, PGLYRP4; B, PGLYRP3; not shown); *, *P*<0.05; **, *P*<0.001; albumin *vs* treated (A); PGRP + O_2_ *vs* no O_2_ (B); PGRP Δ*recA vs* WT (C).

Treatment of bacteria with human recombinant PGRP [4], [5], [7] or paraquat (an O_2_^−^ and H_2_O_2_-inducing positive control) [12], [18] strongly induced intracellular H_2_O_2_ production in both *E. coli* and *B. subtilis*, which was maximal at 15 min (Figure 2A), remained equally high at 30 min, and began to decline after 60 min, likely due to instability of H_2_O_2_ (data not shown). H_2_O_2_ was not induced by albumin (negative control) or diamide (thiol-oxidizing disulfide stress-inducing agent as another control) (Figure 2A).

### ROS are required, but not sufficient for PGRP-induced killing

To determine whether PGRP-induced ROS are required for PGRP-induced killing, we determined the requirement for oxygen for PGRP-induced bacterial killing, as ROS cannot be formed in the absence of oxygen. In the presence of oxygen, PGRP reduced the numbers of *E. coli* and *B. subtilis* by nearly 4 logs in 4 hrs. However, in the absence of oxygen (90% N_2_, 5% H_2_, 5% CO_2_), PGRP did not kill *E. coli*, and under microaerophilic conditions (1% O_2_) PGRP did not kill *B. subtilis* either (Figure 2B). However, under anaerobic or microaerophilic conditions, PGRP was still bacteriostatic for both bacteria. These results show that oxygen is required for PGRP-induced killing, and also indicate additional oxygen-independent antibacterial mechanisms of PGRPs.

Oxidative damage of DNA by ROS greatly contributes to their toxicity, and mutants deficient in the excision or recombinational repair of oxidative DNA lesions are especially sensitive to oxidative stress [12], [14], [17]. Accordingly, a Δ*recA E. coli* mutant was significantly more sensitive to PGRP than WT bacteria (Figure 2C). These results are consistent with the hypothesis that oxidative DNA damage significantly contributes to the bactericidal effect of PGRPs.

**Figure 3:**
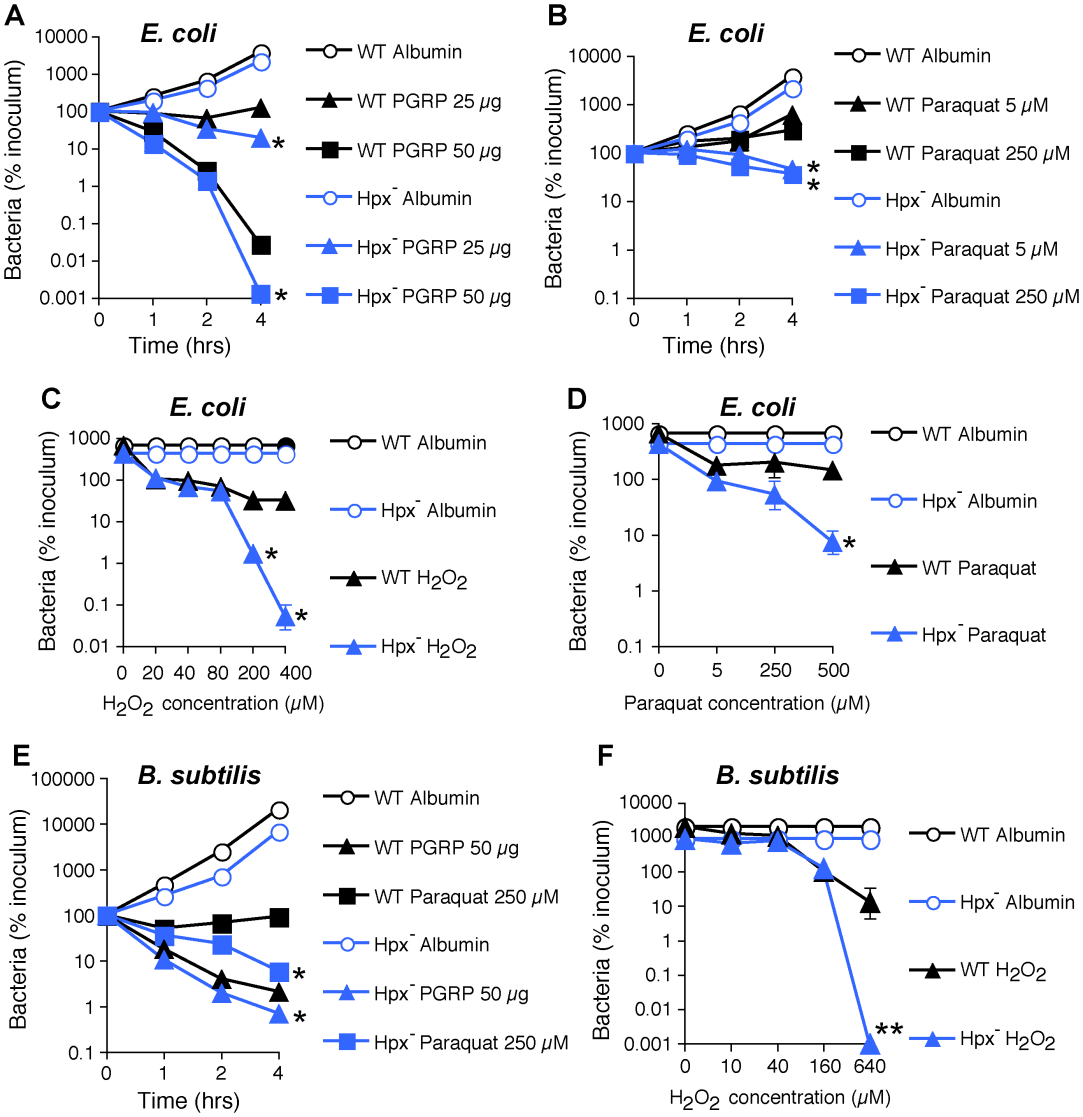
H_2_O_2_ is not sufficient for PGRP killing of bacteria. WT or Hpx^−^ *E. coli* or *B. subtilis* were incubated aerobically with PGRP (A, PGLYRP3; B, PGLYRP3:PGLYRP4), albumin, paraquat, or H_2_O_2_ for 1, 2, or 4 hrs (A, B, E) or 2 hrs (C, D, F) and the numbers of bacteria were determined. The results are means ± SEM of 3 experiments (SEM were within symbols, if not visible); *, *P*<0.05; **, *P*<0.001; WT *vs* Hpx^−^.

To further determine the role of ROS in bacterial killing, we evaluated killing of WT and Hpx^−^ *E. coli* and *B. subtilis* by PGRP, paraquat (which directly induces intracellular H_2_O_2_ production), and exogenously added H_2_O_2_. PGRP readily killed WT and Hpx^−^ *E. coli* and *B. subtilis*, and Hpx^−^ mutants were more sensitive to PGRP killing than WT *E. coli* and *B. subtilis* (Figure 3A, E). However, the concentrations of paraquat (5–250 µM) that induce comparable amounts of H_2_O_2_ production as PGRP (∼1.5 µM H_2_O_2_ induced by 5 µM paraquat, compared with 1.2–2.2 µM H_2_O_2_ induced by PGRP in Figure 2A) were only bacteriostatic and did not kill WT *E. coli* and *B. subtilis* (Figure 3B, E). Although Hpx^−^ mutants were more sensitive to paraquat than WT bacteria, they were still not killed (*E. coli*) or killed inefficiently (*B. subtilis*) by 5–250 µM paraquat (Figure 3B, D, E). Only high concentration of paraquat (500 µM) was bactericidal for Hpx^−^ mutants, but still not for WT bacteria (Figure 3D). Similarly, only very high concentrations of exogenously added H_2_O_2_ (200–640 µM) were bactericidal for Hpx^−^ mutants, but still not for WT bacteria (Figure 3C, F). These results indicate that the amounts of H_2_O_2_ induced by PGRP or by 5–250 µM paraquat (∼2 µM H_2_O_2_, Figure 2A) are not sufficient to kill bacteria.

Altogether, these results demonstrate that ROS are induced by PGRPs and are required for their bactericidal activity, but that physiologically relevant concentrations of ROS induced by PGRPs are not sufficient for bacterial killing. Therefore, these results suggest that other killing mechanisms work together with O_2_-dependent generation of ROS in eliciting the bactericidal activity of PGRP.

### PGRPs induce thiol depletion, which is required, but not sufficient for PGRP killing

**Figure 4:**
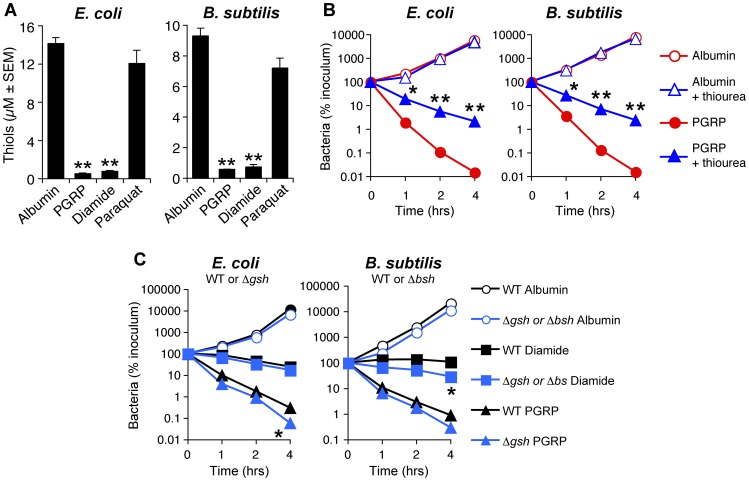
PGRP induces thiol depletion, which is required but not sufficient for bacterial killing. (**A**) *E. coli* or *B. subtilis* were incubated aerobically with albumin (50 µg/ml), PGRP (PGLYRP3, 50 µg/ml), diamide (250 µM), or paraquat (5 µM) for 30 min and intracellular thiols were measured. (**B**) *E. coli* or *B. subtilis* were incubated aerobically with albumin or PGRP (PGLYRP4, 50 µg/ml) without or with thiourea (150 mM, an inhibitor of thiol oxidation), and the numbers of bacteria were determined. (**C**) WT and glutathione-deficient Δ*gshA E. coli* or bacillithiol-deficient Δ*bshC B. subtilis* mutants were incubated aerobically with albumin (50 µg/ml), or diamide (250 µM), or PGRP (PGLYRP4, 50 µg/ml), and the numbers of bacteria were determined. The results are means ± SEM of 3–6 experiments (SEM were within symbols, if not visible); each experiment was repeated once with rMSA and another PGRP with similar results (A, PGLYRP4; B and C, PGLYRP3:PGLYRP4; not shown); *, *P*<0.05; **, *P*<0.001; albumin *vs* treated (A); − *vs* + thiourea (B); WT *vs* Δ*gshA* or Δ*bshC* mutants (C).

We then tested the hypothesis that PGRPs cause thiol (disulfide) stress by inducing depletion of intracellular thiols, because the pattern of gene induction by PGRP was similar to the previously reported pattern of gene induction by diamide (a thiol-depleting electrophile), including activation of genes for the same metal detoxification systems, chaperones, protein quality control, and thiol stress responses [19], [20]. PGRP, similar to diamide, depleted over 90% of intracellular thiols in *E. coli* and *B. subtilis* within 30 min of exposure (Figure 4A), and these low levels of thiols were maintained for at least 2 hrs both in PGRP- and diamide-treated bacteria (data not shown). Paraquat only minimally reduced intracellular thiols (Figure 4A) at a concentration that strongly induced H_2_O_2_ production, comparable to PGRP-induced H_2_O_2_ production (Figure 2A). Altogether, our results show that PGRPs induce both H_2_O_2_ production and thiol depletion, whereas paraquat and diamide selectively induce either H_2_O_2_ production or thiol depletion, respectively.

We next tested the role of intracellular thiols in PGRP killing. Exogenous thiourea (a membrane-permeable thiol that inhibits depletion of thiols and counteracts the effects of thiol and oxidative stress) significantly diminished bactericidal activity of PGRP for both *E. coli* and *B. subtilis* (Figure 4B), consistent with our previous data [7]. These results suggest that depletion of thiols is required for bactericidal activity of PGRPs. We next tested whether depletion of thiols was sufficient for bacterial killing. Diamide, at the concentration that induces similar depletion of thiols as PGRP (Figure 4A), was bacteriostatic, but not bactericidal (Figure 4C). Thus, this level of thiol depletion is not sufficient for bacterial killing.

Glutathione and bacillithiol are the major low molecular weight thiols in *E. coli* and *B. subtilis*, respectively, that protect against oxidative and thiol stress [21]–[23]. Accordingly, glutathione-deficient Δ*gshA E. coli* and bacillithiol-deficient Δ*bshC B. subtilis* mutants had reduced total thiols by ∼45% and ∼30%, respectively (Figure S4). Also, thiol depletion by PGRP or diamide was less efficient in Δ*gshA* and Δ*bshC* mutants (79% and 74% depletion) than in WT bacteria (97% and 90% depletion) (Figure S4), suggesting that glutathione and bacillithiol are major targets of PGRP-induced thiol depletion in *E. coli* and *B. subtilis*, respectively. However, Δ*gshA E. coli* and Δ*bshC B. subtilis* were only somewhat more sensitive to PGRP and diamide than WT strains (Figure 4C), which indicates that these thiols play a modest role in protecting against PGRP and that other cellular thiols in these mutants are still able to maintain nearly sufficient reducing environment in the cytoplasm. Altogether, these results suggest that although thiol depletion likely contributes to bacterial killing, by itself it is not sufficient for strong bactericidal activity.

### PGRP induces increases in intracellular Zn and Cu

**Figure 5:**
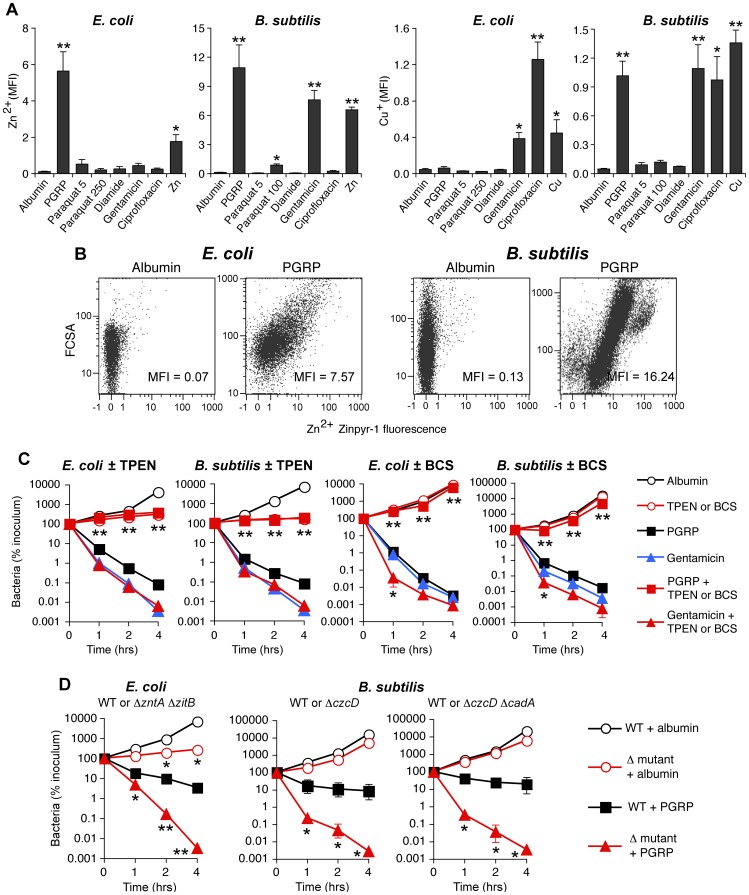
PGRP induces increase in intracellular Zn and Cu, and Zn and Cu are required for killing by PGRP but not by gentamicin. (**A**) *E. coli* or *B. subtilis* were incubated aerobically with albumin (60 µg/ml), PGRP (PGLYRP3:PGLYRP4, 60 µg/ml), paraquat (5, 100, or 250 µM), diamide (250 µM), gentamicin (5 µg/ml), ciprofloxacin (*E. coli*, 100 ng/ml; *B. subtilis*, 1 µg/ml), Zn^2+^ (30 µM), or Cu^2+^ (35 µM), and after 2 hrs free (labile) intracellular Zn^2+^ or Cu^+^ concentration was measured by flow cytometry with Zinpyr-1 and CF4 probes, respectively, and shown as mean fluorescence intensity (MFI). (**B**) Representative dot plots for Zn^2+^ detection are shown (more representative dot plots for Zn^2+^ and Cu^+^ detection are shown in Figure S5). (**C**) *E. coli* or *B. subtilis* were incubated aerobically with albumin (100 µg/ml), PGRP (PGLYRP3 ± TPEN or PGLYRP4 ± BCS, 100 µg/ml), or gentamicin (5 µg/ml), without or with Zn^2+^ chelator TPEN or Cu^+^ chelator BCS (100 µM), and the numbers of bacteria were determined. (**D**) *E. coli* or *B. subtilis* (WT or indicated mutants) were incubated aerobically with albumin or PGRP (PGLYRP4 for *E. coli* or PGLYRP3 for *B. subtilis*, 25 µg/ml), and the numbers of bacteria were determined. The results are means ± SEM of 3–5 experiments (SEM were within symbols, if not visible); experiments in C and D were repeated once with rMSA and another PGRP (PGLYRP4 or PGLYRP3, not shown) with similar results; *, *P*<0.05; **, *P*<0.005 (A) or **, *P*<0.001 (C, D); treated *vs* albumin (A); no TPEN or BCS *vs* with TPEN or BCS (C); or WT *vs* mutant (D).

PGRP treatment highly induced genes for detoxification and efflux of Cu, Zn, and other metals (Figure 1 and Tables 1, S1 and S2). We therefore tested whether treatment with PGRP increased intracellular concentrations of free Zn and Cu (also known as “labile” Zn and Cu, because no metal is truly free in cellular context). Indeed, PGRP induced a large increase in intracellular free (labile) Zn^2+^ in both *E. coli* and *B. subtilis*, based on 60- to 100-fold increase in fluorescence of Zn^2+^-specific membrane permeable Zynpyr-1 probe, measured by flow cytometry (Figures 5A, 5B and S5A). This increase in Zn^2+^ was significant at 30 and 60 min (data not shown) and was maximal at 2 hrs (Figures 5A and S5A). Detection of intracellular Zn^2+^ was completely suppressed by the membrane permeable Zn(II) chelator, TPEN [24] (Figure S5A). PGRP also induced a large increase in intracellular free (labile) Cu^+^ in *B. subtilis*, but not in *E. coli*, based on 20-fold increase in fluorescence of Cu^+^-specific membrane permeable CF4 probe, measured by flow cytometry after 2-hr exposure to PGRP (Figures 5A and S5B). Paraquat and diamide, used at the concentrations that caused similar increase in H_2_O_2_ or depletion of thiols as PGRP, did not induce any increases in intracellular free Zn^2+^ or Cu^+^ (Figure 5A). These results suggest that PGRP-induced increases in intracellular H_2_O_2_ or depletion of thiols are not responsible (or at least not sufficient) for the PGRP-induced increases in intracellular Zn^2+^ and Cu^+^. The slower kinetics of increase in Zn^2+^ and Cu^+^ than accumulation of H_2_O_2_ and depletion of thiols may be related to slower kinetics of transport of exogenous metals into the cell. Thus, these results further suggest that these three effects of PGRPs (oxidative, thiol and metal stress) are independent and do not induce each other.

Antibiotics induced different patterns of changes in intracellular metals than PGRP-induced pattern. Gentamicin treatment led to large increases of both intracellular Zn^2+^ and Cu^+^ in *B. subtilis*, and low, but still significant, increase of Zn^2+^ and a moderate increase of Cu^+^ in *E. coli*. Ciprofloxacin, used here as a known positive control for induction of intracellular Cu^+^ in *E. coli* [25], caused high increase in Cu^+^ in both *E. coli* and *B. subtilis*, but did not lead to increased Zn^2+^ levels (Figures 5A and S5).

### Metal toxicity is required, but not sufficient for PGRP-induced killing

**Figure 6:**
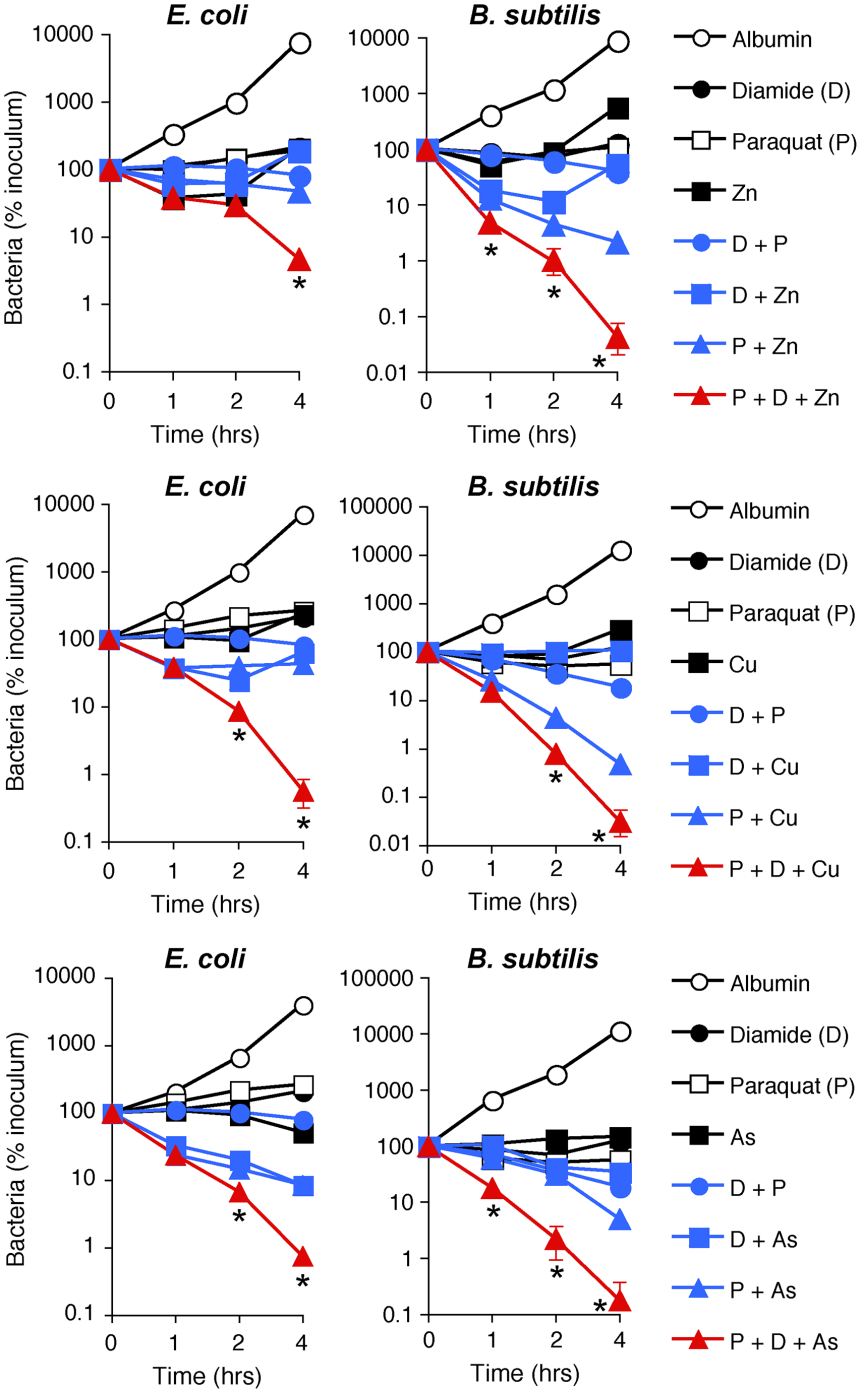
H_2_O_2_ production, thiol depletion, and Zn, Cu, or As synergistically kill *E. coli* and *B. subtilis*. *E. coli* or *B. subtilis* were incubated aerobically with paraquat (50–100 µM), diamide (250 µM), and Zn^2+^ (13 µM *E. coli* or 20 µM *B. subtilis*), or Cu^2+^ (30–35 µM), or As (AsO_2_^−^, 1 mM) individually or together (as indicated), and the numbers of bacteria were determined. The results are means ± SEM of 3–5 experiments (SEM were within symbols, if not visible); *, *P*<0.05 three compounds together *vs* two compounds.

Motivated by the high induction of genes for detoxification of both Cu and Zn (Figure 1 and Tables 1, S1 and S2), and the observed increase in intracellular metal concentrations in both *E. coli* and *B. subtilis* (Figures 5A and S5), we next tested whether Zn^2+^ and Cu^+^ were required for bactericidal activity of PGRPs. Indeed, chelating Zn^2+^ with TPEN completely inhibited the bactericidal activity of PGRP in both *E. coli* and *B. subtilis* (Figure 5C). Chelating Cu^+^ with Cu(I) chelator bathocuprione sulfonate (BCS) [26] also completely inhibited bactericidal activity of PGRP in both *E. coli* and *B. subtilis* (Figure 5C). These effects were selective for PGRP, because TPEN and BCS did not inhibit killing by a bactericidal antibiotic, gentamicin, and BCS even enhanced gentamicin killing at 1 hr (Figure 5C), consistent with the recent report of Cu^+^-mediated induction of antibiotic resistance regulator in *E. coli* [25]. The results with metal chelators, however, need to be interpreted with caution, because chelators are not 100% specific and may chelate to some extent other metals. This could explain the inhibition of PGRP-induced *E. coli* killing by BCS (Figure 5C), when there was no significant PGRP-induced increase in intracellular Cu^+^ in *E. coli* (Figure 5A), because although BCS is a Cu^+^ chelator [26], it can also form dimers with Cu^2+^ and possibly with other divalent metals and chelate them [27]. Our current results are also consistent with our previous data showing that chelating Zn^2+^ with EGTA (whose log stability constant for Zn^2+^ is 12.9) inhibits bactericidal activity of PGRPs, and that 5 µM Zn^2+^ is required for PGRP killing [5]. Our previous results also show that chelating Fe^2+^ with dipyridyl inhibits bactericidal activity of PGRPs [7]. Cu^2+^ and Zn^2+^ at low physiologic concentrations were only bacteriostatic, but not bactericidal (Figure 6), which indicates that at these concentrations Cu^2+^ and Zn^2+^ are not sufficient for bacterial killing.

To further determine which metal ions are the most critical for PGRP-induced killing, we then compared the sensitivity to PGRP and metal killing of WT bacteria and their mutants deficient in various metal efflux and detoxification systems. We show that both *E. coli* Δ*zntA*Δ*zitB* mutant, deficient in two Zn^2+^ efflux systems [28], and *B. subtilis* Δ*czcD* mutants deficient in the Zn^2+^, Cu^2+^, Co^2+^, and Ni^2+^ efflux system [29] were substantially more sensitive to PGRP-induced killing than WT bacteria (Figure 5D). Similarly, Δ*zntA*Δ*zitB* mutant was substantially more sensitive to killing by extracellular Zn^2+^ than WT bacteria (Figure S6A). *E. coli* and *B. subtilis* mutants deficient in Cu efflux and detoxification systems (*E. coli* Δ*copA*Δ*cueO*Δ*cusCFBA* and *B. subtilis* Δ*cadA* and Δ*copZA*) were not more sensitive to PGRP-induced killing than WT bacteria (Figure S6C), and the *E. coli* Δ*copA*Δ*cueO*Δ*cusCFBA* mutant was even more resistant to PGRP killing. Similarly, *E. coli* Δ*copA*Δ*cueO*Δ*cusCFBA* mutant was also more resistant to killing by extracellular Cu^2+^ than WT bacteria (Figure S6A), perhaps because increased intracellular Cu level protects *E. coli* from oxidative Fe toxicity [30], and only at higher concentrations Cu becomes bactericidal. *B. subtilis* Δ*copZA* mutant had similar sensitivity to killing by extracellular Cu^2+^ as WT bacteria, whereas *B. subtilis* Δ*czcD*Δ*cadA* mutant was more sensitive to killing by extracellular Cu^2+^ than WT bacteria (Figure S6B). Higher sensitivity of Zn efflux-deficient than Cu efflux-deficient mutants to PGRP is consistent with high increase of intracellular Zn^2+^ in both PGRP-treated bacteria.

Altogether, these results indicate that Zn^2+^ and Cu^+^ are required for bactericidal activity of PGRPs, and that Zn^2+^ is more important than Cu^+^ for this bactericidal activity, especially in *E. coli*. Our results also indicate that these metals are not required for bactericidal activity of antibiotics. Indeed, PGRPs have the same bactericidal activity towards antibiotic-sensitive bacteria and clinical isolates resistant to multiple antibiotics (Figure S7).

### Synergistic effect of ROS, thiol depletion, and metals is required for bacterial killing

We next tested the hypothesis that production of ROS, depletion of thiols, and metal toxicity have a synergistic bactericidal effect, because these three stress responses were all induced in PGRP-treated bacteria and each was required, but not individually sufficient, for bacterial killing. To induce intracellular ROS production we used paraquat, which is reduced by Complex I to radical cations, which react with O_2_ to generate O_2_^−^, which then generate H_2_O_2_ and then OH^•^ [14], [18]. To induce thiol stress, we used diamide, which directly depletes intracellular thiols by inducing formation of disulfide bonds and S-thiolations (which are disulfide bonds between proteins and low molecular weight thiols, such as glutathione, bacillithiol, and free cysteine) [14], [20], [31]. To induce metal toxicity, we used exogenous Zn^2+^, or Cu^2+^ (which is transported into the cell and reduced to more toxic Cu^+^), or arsenite (AsO_2_^−^).

Indeed, treatment of *E. coli* or *B. subtilis* with the doses of paraquat that induce the amounts of H_2_O_2_ comparable with the amounts of H_2_O_2_ induced by PGRP were not bactericidal (Figure 6). Also, treatment of *E. coli* or *B. subtilis* with the doses of diamide that deplete thiols to a comparable extent as PGRPs were not bactericidal, and low concentrations of Zn^2+^, Cu^2+^, or As (AsO_2_^−^) by themselves were also not bactericidal (Figure 6). Moreover, the combination of any two of these stresses was also not bactericidal (except for a combination of paraquat plus Zn^2+^ or Cu^2+^, which had low killing activity for *B. subtilis*). However, when all three stress conditions were simultaneously imposed, the resulting combination was strongly bactericidal for both *E. coli* and *B. subtilis*, although Zn^2+^ was less efficient in *E. coli* than in *B. subtilis* (Figure 6). These results validate our hypothesis and show that ROS production, thiol depletion, and metal toxicity act synergistically to kill bacteria.

**Figure 7:**
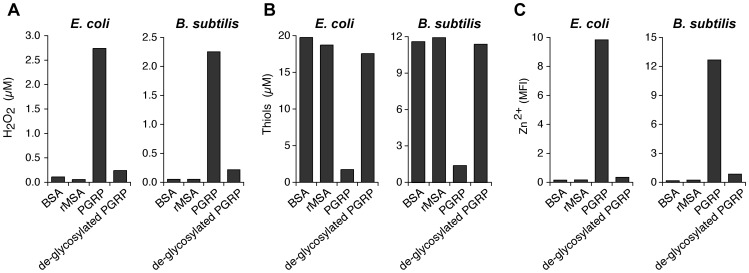
De-glycosylation abolishes the ability of PGRP to induce intracellular production of H_2_O_2_, depletion of cellular thiols, and increases in intracellular Zn^2+^. *E. coli* or *B. subtilis* were incubated with 50 µg/ml of bovine serum albumin (BSA), recombinant mouse serum albumin (rMSA), PGRP (mock treated), or de-glycosylated PGRP, and assayed for H_2_O_2_ (**A**), thiols (**B**), and intracellular free (labile) Zn^2+^ (**C**) as described in Figures 2, 4, and 5, respectively. The results are averages of duplicates from one experiment with PGLYRP3 (shown), which was repeated once with PGLYRP4 with similar results (not shown).

To further verify that oxidative, thiol, and metal stress are responsible for the bactericidal activity of PGRPs, we abolished bactericidal activity of PGRP by de-glycosylation, which we previously showed to be required for bactericidal activity of PGRPs for both Gram-positive and Gram-negative bacteria [4], [5]. De-glycosylation abolished 90–95% of the ability of PGRP to induce (i) intracellular production of H_2_O_2_ (Figure 7A), (ii) depletion of cellular thiols (Figure 7B), and (iii) increases in intracellular Zn^2+^ (Figure 7C) in both *E. coli* and *B. subtilis*. These results further validate the requirement of oxidative, thiol, and metal stress for the bactericidal activity of PGRPs.

## Discussion

Analysis of the global transcriptional responses of both *E. coli* and *B. subtilis* to PGRP revealed stress responses involving increased production of H_2_O_2_, depletion of thiols, and increases in intracellular Zn^2+^ and Cu^+^, which were also verified by direct measurements. Using selective chemical treatments (paraquat to generate ROS, diamide to oxidize thiols, and exogenous metal ions) and specific inhibitors, we demonstrated that ROS production, thiol depletion, and increased intracellular Zn^2+^ or Cu^+^ are all required, but individually are not sufficient, for bacterial killing, and that combined action of oxidative, thiol, and metal stress kills bacteria.

PGRP treatment induced oxidative stress through rapid induction of H_2_O_2_ production. Oxidative stress results from excessive production of ROS (O_2_^−^, H_2_O_2_, and HO^•^). Both O_2_^−^ and H_2_O_2_ oxidize solvent-exposed [4Fe-4S] enzyme clusters, causing release of Fe and cluster collapse to inactive [3Fe-4S]^+^. O_2_^−^ and H_2_O_2_ also inactivate mononuclear iron enzymes by oxidizing Fe-coordinating cysteines or by replacing Fe^2+^ with Zn^2+^ [16], [21], [32]–[34]. Moreover, H_2_O_2_ reacts with Fe^2+^ to generate HO^•^ via Fenton reaction. HO^•^ is the most reactive and most toxic ROS and it irreversibly damages DNA, proteins, and other organic molecules [14], [17].

PGRP treatment also depleted over 90% of cellular thiols. Thiol stress results from oxidation of thiols, which maintain the redox state in the cells and protect from oxidative damage. Oxidative and thiol stress not only directly damage cells, but also release Fe from proteins, increase intracellular concentration of Zn and Cu, and increase toxicity of most metals [19], [21], [35]–[37]. Thiols bind free metal ions and protect cells from metal toxicity [38], and for this reason thiol stress induces the same genes for metal detoxification and protein refolding and repair [19], [20], [31] as the genes induced by PGRP (Tables 1, S1, and S2).

PGRP treatment also induced a drastic increase in intracellular free (labile) Zn^2+^ in both *E. coli* and *B. subtilis* and intracellular free (labile) Cu^+^ in *B. subtilis* (but not *E. coli*), which is the likely reason for increased expression of metal detoxification and efflux genes. These increases in free metals are required for PGRP toxicity, because chelating intracellular Zn^2+^ with TPEN (Figure 5C) or extracellular Zn^2+^ with EGTA [5], or chelating Cu^+^ with BCS (Figure 5C) or Fe^2+^ with dipyridyl [7] also inhibits bacterial killing by PGRP. Zn^2+^ seems the most important for PGRP killing, as revealed by the highest sensitivity of Zn^2+^ efflux mutants to PGRP killing (Figure 5D). However, the increased concentrations of metals alone that are induced by PGRP are not sufficient for bacterial killing.

The origins of metal toxicity are complex. Zn, a redox-inert metal, is more abundant in the cytosol than Cu and at low concentrations it may protect bacteria from oxidative and thiol stress, likely by binding to thiols and preventing their further oxidation [39]. However, high levels of Zn are toxic and up-regulate the expression of genes for Zn efflux (*zntA* in *E. coli* and *czcD* and *cadA* in *B. subtilis*, also observed in our arrays). Zn toxicity, similar to Cu, results in part from inactivation of solvent-exposed Fe-S clusters; and although this activity of Zn^2+^ is lower than Cu^+^ [40], it is likely compensated by higher concentrations of Zn^2+^ than Cu^+^. In oxidative stress, Zn^2+^ also inactivates mononuclear enzymes by replacing Fe^2+^ in their active sites [32]. Cu is toxic because it causes loss of Fe from solvent-exposed Fe-S clusters, which inactivates enzymes, and also because this release of Fe makes it available for enhanced production of HO^•^ via Fenton reaction [14], [21], [35]–[37], [41]–[44]. Cu also causes thiol oxidation and sulfhydryl depletion, which contribute to thiol stress and protein damage [21], [35], [37], [42]. Fe toxicity results primarily from generation of HO^•^, which damages DNA, proteins, and lipids [14], [29]. HO^•^ is induced by PGRPs and chelating intracellular Fe with dipyridyl inhibits both HO^•^ production and PGRP killing [7].

Many of the genes induced by PGRPs reflect direct or indirect bacterial responses to the resulting oxidative, thiol, and metal stress. The genes for repair of damaged proteins and DNA and Ihf-regulated genes (which help to maintain DNA architecture) are induced because ROS oxidize proteins and nucleic acids, because oxidation of thiols damages proteins, and because increased concentrations of intracellular metals also damages proteins [14], [16], [17], [19], [21], [33], [34], [41]–[44]. Genes for transition to fermentation and anaerobic growth (e.g., members of Fnr regulon in *E. coli*) are a likely attempt to reduce the use of oxygen to limit further production of ROS. Genes for energy generation are induced because of possible oxidative damage to respiratory chain enzymes and because a decrease in membrane potential [7] may cause a decrease in ATP production by membrane potential-driven ATP synthase [45], [46]. This is also the likely reason why bacteria down-regulate genes for high energy-requiring non-essential processes, such as motility, which are controlled by CpxRA [8], one of the regulators of envelope stress response activated by PGRP [7].

The genes for methionine and histidine synthesis may be induced for several reasons. These amino acids are essential metal-binding components abundant in metal detoxification proteins, e.g., methionine shuttle is used for Cu efflux and histidine is used for coordination of metals in metal detoxification proteins, such as CusA and CopA Cu efflux and AraA and ArsD As efflux transporters [47]–[49]. Also, histidine shares biosynthetic intermediates with nucleotides, whose synthesis is needed to repair damaged DNA. Moreover, likely oxidation of the thiol group in homocysteine may deplete this methionine biosynthesis intermediate. Methionine is also needed for initiation of translation and DNA replication, and methionine synthase is highly sensitive to thiol stress [50].

The genes for Fe-S cluster assembly (*isc* in *E. coli*) are likely induced due to the damage to Fe-S clusters by oxidative, thiol, and metal stress, and most likely Cu^+^-induced release of Fe^2+^ from Fe-S clusters. Cu^+^ also damages Isc proteins, which may further contribute to the induction of *isc* genes. Damage to DNA could be either direct by Cu^+^, or more likely by Cu^+^-induced release of Fe^2+^ from Fe-S clusters and Fe-driven enhancement of HO^•^ production from H_2_O_2_ [21]. This mechanism is supported by the ability to inhibit PGRP killing by chelating either Fe^2+^ with dipyridyl [7] or Cu^+^ with BCS (Figure 5C). Concurrently bacteria down-regulate the expression of genes for Fe uptake, which also suggests an increase in cytoplasmic free Fe^2+^, likely due to release of Fe^2+^ from Fe-S clusters, caused by oxidative and thiol stress and Cu^+^. Down-regulation of Fe uptake is controlled by the envelope stress response regulator CpxRA, which is activated by PGRPs [7], and by increased Cu and Zn [8], [51]–[53].

How do PGRPs induce oxidative, thiol, and metal stress in bacteria? PGRPs have a specific peptidoglycan-binding grove that binds disaccharide-pentapeptide fragment of peptidoglycan [2], [3], [54], [55]. However, this PGRP-binding site on peptidoglycan is not easily accessible on the surface of Gram-positive bacteria, because of extensive peptidoglycan cross-linking and its substitution with polysaccharides and proteins. Thus, in Gram-positive bacteria PGRPs preferentially bind to the separation sites of the newly formed daughter cells, created by dedicated peptidoglycan-lytic endopeptidases, which separate daughter cells after cell division. We assume that these cell-separating endopeptidases expose PGRP-binding muramyl peptides, because PGRP bound to bacteria co-localizes with cell-separating endopeptidases and PGRPs do not bind to other regions of the cell wall with highly cross-linked peptidoglycan [7]. This localization is necessary for bacterial killing, because mutants that lack these endopeptidases and do not separate after cell division (Δ*lytE*Δ*lytF B. subtilis*) do not bind PGRPs and are not killed by PGRPs [7]. In Gram-negative bacteria, PGRPs bind uniformly to the entire outer membrane [7], which is composed of lipopolysaccharide (LPS) and covers a thin peptidoglycan layer. This is possible, because in addition to binding peptidoglycan, PGRPs also bind LPS using binding sites outside the peptidoglycan-binding groove [56], [57]. This binding to bacterial envelope is required for PGRP killing, because exogenous peptidoglycan or LPS inhibit PGRP killing of Gram-positive or Gram-negative bacteria, respectively, by blocking peptidoglycan or LPS binding sites on PGRP [4], [5], [56]. It is not known whether after binding to LPS in Gram-negative bacteria PGRPs also bind to peptidoglycan, located in the periplasmic space beneath the outer membrane. In both Gram-positive and Gram-negative bacteria, after binding to peptidoglycan or LPS, PGRPs do not enter the cytoplasm [7], but probably form oligomeric ribbon-like structures [2], [55] and induce envelope stress by activating stress response two component systems, CpxRA in *E. coli* and CssRS in *B. subtlis*, which are typically activated by misfolded or aggregated proteins exported from the cells [3], [7], [58]. This activation ultimately results in membrane depolarization, inhibition of all biosynthetic reactions, and cell death [7]. However, the exact initial mechanism through which PGRPs activate envelope stress response and oxidative, thiol, and metal stress is unknown, as this mechanism is also unknown for other envelope stressors [8], and is currently under investigation. Furthermore, based on induction of multiple stress response regulons by PGRP (Tables 1, and S1, S2, S3, S4) and on incomplete resistance of Δ*cpxRA* and Δ*cssRS* mutants to PGRP [7], it is likely that PGRPs activate other stress sensors that induce these multiple stress responses.

Other investigators previously proposed that oxidative stress is involved in killing of *E. coli* by antibiotics [9], [10]. However, recent results do not support this conclusion [12], [13] and are consistent with our results. Our data clearly indicate that the mechanisms of killing by PGRPs and antibiotics are different for the following reasons. (i) PGRPs kill bacteria resistant to multiple antibiotics (Figure S7) [4]. (ii) PGRP killing requires O_2_ and PGRPs do not kill anaerobically (Figure 2B), whereas many antibiotics kill both aerobically and anaerobically [12], [13]. (iii) PGRPs very strongly induce peroxide-responsive genes (e.g. the OxyR regulon in *E. coli*) indicating endogenous H_2_O_2_ production, but antibiotics do not (Tables S1 and S2) [12]. (iv) PGRPs strongly induce H_2_O_2_ production in bacteria (Figure 2A), but antibiotics do not [12]. (v) Δ*recA* mutant is more sensitive than wild type strain to PGRPs (Figure 2B), but not to antibiotics [12]. (vi) PGRP-induced killing is inhibited by chelating Zn^2+^ or Cu^+^, whereas killing by antibiotics is not affected by chelating Zn^2+^ and is enhanced by chelating Cu^+^ (Figure 5C). These results are consistent with induction of the antibiotic resistance regulator MarR by CpxRA [59] and by Cu^+^ [25], which are induced by both PGRP and antibiotics. However, MarR confers resistance only to antibiotics [25], but not to PGRP. (vii) The patterns of gene expression induced in *E. coli* and *B. subtilis* by bactericidal concentrations of PGRP and by gentamicin are different: more than half of the top 100 genes strongly induced by PGRPs are not induced by gentamicin, e.g., genes for oxidative stress, energy production, Fe-S cluster repair and assembly, Fe-S-containing enzymes (e.g., *edd*), amino acid synthesis, and other stress responses. (viii) We could prevent bacterial killing by cell wall synthesis-inhibiting antibiotics, but not by PGRPs, using hyperosmotic medium [7], which should not happen if the main mechanism of killing by these antibiotics was due to oxidative stress and was the same as for PGRPs. (ix) Antibiotics selectively inhibit one biosynthetic reaction and other biosynthetic reactions are not inhibited for several hours until bacteria die, whereas exposure to PGRPs results in simultaneous and rapid inhibition of all biosynthetic reactions in bacteria [7].

PGRPs, bactericidal innate immunity proteins, by combining oxidative stress with thiol depletion and release of intracellular metals, have evolved a powerful antibacterial defense strategy. This strategy is consistent with recent evidence that phagocytic cells, upon phagocytosis of bacteria, in addition to oxidative killing, pump Cu and Zn into phagolysosomes to enhance bacterial killing [41]–[44], [60]. Indeed, the most abundant PGRP, PGLYRP1, is present in neutrophil, eosinophil, and macrophage granules [1], [56], [61]–[64], and other PGRPs (PGLYRP2, PGLYRP3, and PGLYRP4) are produced on the skin and mucous membranes, and in sweat, sebum, and saliva [1], [4], [5]. These body secretions also contain significant amounts of Cu and Zn [5], which is consistent with the requirement for Zn (Figure 4B) [5], Fe [7], and Cu (Figure 4B) for bactericidal activity of PGRPs. In response to PGRPs bacteria up-regulate expression of Cu and Zn exporters (CopA, ZntA, CadA, and CzcD). However, PGRPs defeat this bacterial Cu and Zn defense, because PGRP-induced oxidative, thiol, and metal stress likely damage respiratory chain enzymes and depolarize bacterial membranes [7], which likely reduces ATP production and proton motive force needed to drive bacterial Cu and Zn efflux. Furthermore, because Cu tolerance increases bacterial virulence [41]–[44], targeting Cu tolerance will both increase bacterial killing and decrease bacterial virulence, which should additionally improve host defense against infection.

*In vivo* PGRPs are present at concentrations similar to the concentrations used in our experiments: PGLYRP1 is present in milk at 120 µg/ml [65] and in polymorphonuclear leukocytes' granules at 2.9 mg/10^9^ cells [64], PGLYRP2 is present in serum at 100 µg/ml [66], [67], and PGLYRP3 and PGLYRP4 are secreted on mucous membranes, likely reaching similar local concentrations [1], [4]. In this study we investigated the mechanism of bactericidal activity of PGRPs *in vitro*, but the following evidence indicates that PGRPs also have antibacterial activity *in vivo*: (i) local application of PGRPs into upper respiratory tract protects mice against lung infection [4], [58]; (ii) *Pglyrp1* ^−/−^ mice are more sensitive to some infections than wild type mice [62]; (iii) neutrophils from *Pglyrp1* ^−/−^ mice are less efficient in bacterial killing than neutrophils from wild type mice [62]; (iv) PGRPs protect zebrafish embryos from bacterial infections [68]; (v) PGRPs are required for maintenance of normal intestinal microbiome in mice [69]; and (vi) PGRPs also have several anti-microbial and microbiome-regulating functions in invertebrates [3]. Our results indicate that PGRPs have bactericidal activity in an aerobic environment, which is consistent with the highest expression of PGRPs in phagocytic cells and on the skin and mucous membranes, especially in the mouth, throat, esophagus, and salivary glands [1]–[4], [56], [61]–[64], [69]. Lower PGRP expression in the stomach and small and large intestine is again consistent with their bactericidal activity in an aerobic environment, although anaerobically PGRPs are still bacteriostatic. Bactericidal activity of PGRPs both *in vitro* and *in vivo* is enhanced by antimicrobial peptides [5], [58], also expressed in phagocytic cells and on mucous membranes and skin, which likely further strengthens antibacterial defenses of the host.

In conclusion, innate immunity proteins, PGRPs, induce oxidative, thiol, and metal stress in *E. coli* and *B. subtilis*, which act synergistically to kill bacteria. Because this bactericidal mechanism differs from killing by antibiotics and because PGRPs kill antibiotic-resistant bacteria, synergistic targeting of oxidative, thiol, and metal stress can be used for the development of new approaches to treatment of antibiotic resistant bacteria.

## Materials and Methods

### Materials

Bacterial strains are listed in Table S5. Disruption of *Bacillus* genes was achieved by transformation with PCR products to amplify DNA fragments flanking each target gene and an intervening antibiotic cassette as previously described [70]. Human PGRPs (PGLYRP3, PGLYRP4, and PGLYRP3:PGLYRP4 heterodimer) were expressed in S2 cells and purified as previously described [4], [5] in a buffer containing 10 mM TRIS (pH 7.6), with 150 mM NaCl, 10 µM ZnSO_4_, and 10% glycerol. The experiments were done using PGLYRP3, PGLYRP4, and/or PGLYRP3:PGLYRP4 (as indicated in Figure legends and Table footnotes), and all key experiments were performed with at least two PGRPs with similar results. Note that when expressed individually, PGLYRP3 and PGLYRP4 form disulfide-linked homodimers, and when co-expressed in the same cells, they form disulfide-linked PGLYRP3:PGLYRP4 heterodimers [4]. For some experiments PGRP was de-glycosylated by treatment with 0.67 units of N-glycosidase/µg PGRP (PNGase F from *Elizabethkingia miricola*, Sigma) for 2 hr at 37°C, and we verified that this treatment abolished PGRP's bactericidal activity for *E. coli* and *B. subtilis*, as previously described [4], [5]. For non-de-glycosylated PGRP in these experiments, PGRP was similarly incubated in the same buffer, but without PNGase. Purified bovine serum albumin (BSA, Sigma) was used as a negative control, and key experiments were repeated with recombinant mouse serum albumin (rMSA) as an additional control, which was cloned, expressed, and purified by the same methods as PGRPs, as described [7], with similar results, as indicated in figure legends. Paraquat (methyl viologen) was from Acros Organics, Zinpyr-1 and TPEN were from Santa Cruz. Bathocuprione disulfonate (BCS), CCCP (carbonyl cyanide 3-chlorophenyl-hydrazone), ciprofloxacin, diamide, gentamicin, and other reagents were from Sigma-Aldrich, unless otherwise indicated. Arsenite (AsO_2_^−^) was prepared fresh from arsenic trioxide at pH 8.2; CuSO_4_ was used as Cu^2+^, and ZnSO_4_ as Zn^2+^.

### Gene expression arrays

Overnight bacterial cultures were diluted 1∶100 in LB, grown aerobically with 250 rpm shaking to OD_660_ = 0.1–0.3, suspended in fresh warm medium (*E. coli* MG1655 at OD_660_ = 0.3 or *B. subtilis* 168 at OD_660_ = 0.1), and incubated aerobically with 100 µg/ml albumin (control), or 100 µg/ml PGRP (human recombinant PGLYRP4), or 5 µg/ml gentamicin for 30 min, or with 800 µM CCCP for 15 min, in 2 ml of 5 mM TRIS (pH 7.6) with 150 mM NaCl, 5 µM ZnSO_4_, with addition of 2% of 100% LB (*E. coli*), or in 1 ml of TRIS-Schaeffer medium with 0.05% NH_4_Cl, 5 µM ZnSO_4_, 0.2% glucose, with addition of 2% of 100% LB (*B. subtilis*) at 37°C with 250 rpm shaking (these optimum incubation times and concentrations for induction of stress response genes were determined in preliminary experiments using qRT-PCR). Because 5 µM Zn^2+^ is required for bactericidal activity of PGRP and corresponds to the average concentration of Zn^2+^ found in saliva, sweat, and other body fluids, where PGRPs are present [5], we confirmed that Zn^2+^ is not depleted or increased by additions of our proteins and bacteria, by measuring the concentration of free Zn^2+^ in our incubation mixtures at the initiation of our experiments (time 0) using Zn^2+^-specific probe, Zinpyr-1, and fluorescence spectroscopy (with Molecular Devices Gemini EM Spectrofluorometer). Our incubation mixtures containing 100 µg/ml of either PGRP (PGLYRP3 or PGLYRP4) or recombinant mouse albumin, and with or without addition of bacteria, all contained similar amounts of free Zn^2+^ (4.1–4.4 µM). Moreover, substituting the addition of 5 µM free Zn^2+^ with the addition of 25 µM Zn^2+^ plus 20 µM EDTA (a divalent cation chelator with high affinity for Zn^2+^, log dissociation constant 16.6) yielded the same concentrations of free Zn^2+^ as in our reaction mixture without EDTA, measured by Zinpyr-1 fluorescence. Based on these results we concluded that addition of our control protein or PGRPs and/or bacteria does not substantially change the free Zn^2+^ concentration in our experiments.

To obtain RNA from each culture, bacteria were harvested and RNA was extracted using Ambion RiboPure-bacteria RNA extraction kit according to the manufacturer's instructions. For *B. subtilis*, before RNA extraction, bacteria were disrupted by shaking with Zirconia beads. cDNA was synthesized with random hexamer primers, fragmented, labeled with terminal transferase and biotin, and hybridized to whole genome Affymetrix *E. coli* Genome 2.0 Array GPL3154 or custom whole genome Affymetrix 900513 GeneChip *B. subtilis* Genome Array using Affymetrix Hybridization Oven 640 and Affymetrix GeneChip Fluidics Station 450 and protocols provided by Affymetrix GeneChip Technical Manual. Scanning and data extraction were done using Affymetrix GeneChip Scanner 3000 and protocols provided by Affymetrix GeneChip Technical Manual. cDNA synthesis, labeling, hybridization, and scanning were performed at the Genomic and RNA Profiling Core facility, Baylor College of Medicine, Houston, TX. The entire experiment was repeated 3 times both for *E. coli* and *B. subtilis*.

Hybridization intensity data signals were analyzed, normalized, and corrected for batch effect using Affymetrix GeneChip Command Console Software. Signal average, noise average, scaling factor, % present, and % absent were calculated for each probe. From this analysis, for *E. coli*, signal intensity of ≥39 was calculated as reliable expression, and using this cutoff, 5,531 probes were classified as present out of total 10,208 probes on the array. For *B. subtilis*, signal intensity of ≥78 was calculated as reliable expression, and using this cutoff, 3,355 probes were classified as present out of total 5,039 probes on the array. The probes were classified as expressed when at least one experiment in one group showed the signal intensity ≥39 for *E. coli* or ≥78 for *B. subtilis*. Signal intensities from 3 experiments were used to calculate fold increases or decreases in gene expression between treated and control groups, with signal intensity of 39 for *E. coli* or 78 for *B. subtilis* used as a minimum intensity (i.e., for these calculations all signal intensities of <39 for *E. coli* and <78 for *B. subtilis* were replaced with 39 or 78, respectively). The fold changes in gene expression were calculated using the formula: intensity in treated group/geometric mean of intensity in control (albumin) groups, and reported as means ± SEM in Tables S1, S2, S3, S4. This method yields conservative fold increases or decreases in gene expression and avoids erroneous and unrealistically large fold changes in gene expression, which would have been obtained if signal intensities below the reliable expression thresholds were used for these calculations. Transformed Ln(signal intensity) values were used for direct statistical comparisons of expression signals between treated and control (albumin) groups. We deposited all whole genome expression arrays data in NCBI GEO (accession numbers GSE44211 and GSE44212).

We also compared by hierarchical cluster analysis [71] our whole genome expression results with published data on *E. coli* exposed to H_2_O_2_ [72] and Zn (NCBI GEO GSE26187), and on *B. subtilis* exposed to vancomycin [73], diamide [19], H_2_O_2_ [74], and Zn [29].

### Annotation of gene functions and regulation

The functions of genes, gene operons, and gene regulons were annotated using the following web databases: for *E. coli*: PrFEcT (http://www.prfect.org/index.php?option=com_content&view=frontpage&Itemid=1), GenExpDB (http://www.prfect.org/index.php?option=com_wrapper&view=wrapper&Itemid=38), and RegulonDB (http://regulondb.ccg.unam.mx/index.jsp); and for *B. subtilis*: SubtilisWiki (http://subtiliswiki.net/wiki/index.php/Main_Page) and SubtiWiki (http://subtiwiki.uni-goettingen.de/).

### qRT-PCR

*E. coli* or *B. subtilis* (300 µl each) were incubated with albumin (control), PGRP, gentamicin, or CCCP, and RNA was extracted as described above for gene expression arrays. The amounts of mRNA were measured using quantitative reverse transcription real-time PCR (qRT-PCR) as previously described [7], [63]. cDNA was synthesized from 100 ng of RNA using RT^2^ PCR Array First Strand Kit (Qiagen/SA Biosciences). Gene expression was quantified by qRT-PCR using the ABI 7000 Sequence Detection System with 1 cycle 10-min at 95°C and 40 cycles 15 sec at 95°C and 1 min at 60°C using Qiagen/SA Biosciences SYBR Green Master Mix and the gene-specific primers (listed in Table S6) or common primers for 16S rRNA from all Eubacteria (ACTCCTACGGGAGGCAGCAGT and ATTACCGCGGCTGCTGGC) as a housekeeping gene. For each gene, ΔCt was calculated followed by normalization to the housekeeping gene, followed by calculation of ΔΔCt for each gene: ΔΔCt = ΔCt1−ΔCt2, where ΔCt1 is the PGRP- or gentamicin- or CCCP-treated bacteria and ΔCt2 is albumin-treated bacteria. This calculation gives the fold increase in expression of each gene in PGRP- or gentamicin- or CCCP-treated bacteria *versus* albumin-treated bacteria. The entire experiment was repeated 3 times both for *E. coli* and *B. subtilis*.

### Assays for H_2_O_2_ and thiols

To measure production of H_2_O_2_, Hpx^−^ strains Δ*katG*Δ*katE*Δ*ahpCF E. coli* and Δ*katA*Δ*ahpCF B. subtilis* were used, which allow accumulation and measurement of H_2_O_2_ [12], [15]–[17]. Bacteria (50 µl) were incubated as for gene expression arrays with albumin (control), PGRP, paraquat, or diamide (at concentrations given in Results), for 15–120 min (15 min was the optimum time for the highest induction of H_2_O_2_, determined in preliminary experiments), and total amount of H_2_O_2_ was determined using fluorescent Amplex Red Hydrogen Peroxide/Peroxidase Assay Kit (InVitrogen/Molecular Probes) according to the manufacturer's instructions. To measure depletion of thiols, 50 µl of *E. coli* MG1655 or *B. subtilis* 168 were incubated as above for H_2_O_2_ production for 30–120 min (30 min was the optimum time for depletion of thiols, determined in preliminary experiments), and the total amount of reduced thiols was determined using fluorescent Measure-iT Thiol Assay Kit (InVitrogen/Molecular Probes) [35] according to the manufacturer's instructions.

### Bactericidal assay

For bactericidal assays, overnight bacterial cultures were diluted 1∶100 in LB, grown aerobically at 37°C with 250 rpm shaking to OD_660_ = 0.1, suspended at ∼2–4×10^6^ bacteria/ml in 50 µl of fresh warm medium, for *E. coli* in 5 mM TRIS (pH 7.6) with 150 mM NaCl, 5 µM ZnSO_4_, 5% glycerol, with addition of 2% of 100% LB [5] or for *B. subtilis* in TRIS-Schaeffer medium with 0.05% NH_4_Cl, 5 µM ZnSO_4_, 5% glycerol, 0.2% glucose, with addition of 2% of 100% LB [4], [7], incubated at 37°C aerobically with 250 rpm shaking, and the numbers of bacteria were determined by colony counts [4]. Assays on killing under anaerobic conditions were done in the same medium in complete absence of oxygen (90% N_2_, 5% H_2_, 5% CO_2_) for *E. coli* or under microaerophilic conditions (1% O_2_) for *B. subtilis* (because *B. subtilis* grows very poorly under strict anaerobic conditions) in Anaerobe Systems AS-580 Anaerobic Chamber for growing the cultures before the assay, during the killing assay, and during incubation of plates for colony counts. Bactericidal activity is defined as an at least 100-fold decrease in the number of inoculated bacteria in 4 hrs.

### Assays for intracellular Zn^2+^ and Cu^+^

*E. coli* MG1655 or *B. subtilis* 168 were prepared and incubated aerobically as for bactericidal assays at ∼2×10^7^ bacteria/ml for 0.5, 1, 2, or 4 hrs with albumin, PGRP, paraquat, diamide, gentamicin, ciprofloxacin, Zn^2+^, or Cu^2+^ (at concentrations indicated in Results), and without or with 100 µM TPEN for Zn^2+^ assay or with 8 µM (*E. coli*) or 2 µM (*B. subtilis*) CuSO_4_ for Cu^+^ assay. Bacteria were washed, incubated with Zinpyr-1 (dissolved in DMSO with 20% Pluronic F-127, 5 µM final concentration) for 15 min at 37°C for free (labile) intracellular Zn^2+^ determination [24], or with Copperfluor-4 (CF4, dissolved in DMSO, 2 µM final concentration) for 15 min at 37°C for Cu^+^ determination. CF4 is a 4^th^ generation membrane permeable fluorescent probe that specifically detects free (labile) intracellular Cu^+^, with improved fluorescent signal, compared with previous CS1–CS3 probes [75]. Bacteria were washed and analyzed by flow cytometry using MACSQuant (Miltenyi) flow cytometer and FITC excitation and emission settings. The maximum increases in Zinpyr-1 and CF4 fluorescence were seen after 2 hrs of incubation and these results are reported as mean fluorescence intensity (MFI) ± SEM. Representative dot plots are also shown in some figures.

### Statistical analyses

Quantitative results are presented as means ± SEM, with statistical significance of the differences between groups determined by the two-sample one-tailed Student's *t*-test using Microsoft Excel; *P*≤0.05 was considered significant. The *n* and *P* values are indicated in the figures and tables. Some gene expression results are presented as heat maps generated using Java TreeView. For microarray data statistical significance of differences in gene expression was also analyzed by calculating *P* values using two-sample two-tailed Student's *t*-test, followed by calculation of π_0_(λ) and then FDR (false discovery rate) *q* values, with significance threshold of *q*≤0.05, as described [76].

## Supporting Information

Figure S1 ***E. coli*** **genes up-regulated or down-regulated more than 3 times by PGRP, gentamicin, or CCCP.** The results are heat-maps of mean ratios of the gene expression signals in PGRP-, gentamicin-, or CCCP-treated to control albumin-treated bacteria determined by whole genome expression arrays from 3 experiments (performed as described in Tables S1 and S3), with maximum and minimum signal intensity set at +10 and −10, and arranged from the highest to the lowest fold induction in PGRP-treated group. The mean expression data, the significance of differences, and the gene functions and regulators of top up- and down-regulated genes are shown in Tables S1 and S3 (all data deposited in NCBI GEO under the accession number GSE44211).
(TIF)

Figure S2 ***B. subtilis*** **genes up-regulated or down-regulated more than 3 times by PGRP, gentamicin, or CCCP.** The results are heat-maps of mean ratios of the gene expression signals in PGRP-, gentamicin-, or CCCP-treated to control albumin-treated bacteria determined by whole genome expression arrays from 3 experiments (performed as described in Tables S2 and S4), with maximum and minimum signal intensity set at +10 and −10, and arranged from the highest to the lowest fold induction in PGRP-treated group. The mean expression data, the significance of differences, and the gene functions and regulators of top up- and down-regulated genes are shown in Tables S2 and S4 (all data deposited in NCBI GEO under the accession number GSE44212).
(TIF)

Figure S3 **Hierarchical cluster display of 605 (** ***E. coli*** **) and 594 (** ***B. subtilis*** **) most up-regulated or down-regulated genes in PGRP, gentamicin, CCCP, H_2_O_2_, Zn, vancomycin, or diamide treated bacteria.** Log_2_-transfomed gene expression data were clustered based on expression level. **(A)** ***E. coli*** **:** **Cluster I** contains genes induced mainly by PGRP (e.g., OxyR-induced: *gor*, *hemH*, *yaaA*, *ahpF*, *katG*, and some metal-stress response genes: *arsC, ndh*). **Cluster II** contains genes strongly induced by PGRP and also induced by gentamicin and CCCP (e.g., energy acquisition genes and other stress genes). **Cluster III** contains genes induced by PGRP and gentamicin, including protein and RNA quality control (*rttR, clpB, htpG, dnaK*), Fe-S cluster repair (*iscS, iscR*), and some oxidative and metal stress genes (*oxyS, arsB, arsR*). **Cluster IV** contains genes induced by PGRP and CCCP (mostly genes for alternative energy sources). **Cluster V** contains genes induced by PGRP, CCCP, and Zn (genes for several transporters). **(B)** ***B. subtilis*** **: Cluster I** contains genes induced by PGRP only (e.g., *uvrA, addA* for DNA repair, and *cysS, yrk* operons), genes induced by PGRP and vancomycin (*his* operon), and genes induced by PGRP and gentamicin (*ycgM, ycgN* for proline utilization, and *pur* operon for purine synthesis). **Cluster II** contains genes common to several treatments, including some of the ROS-induced PerR regulon-controlled genes (*mrgA, katA, ykvW = zosA*) and CymR-rgulated genes for obtaining cysteine and methionine. **Cluster III** contains several metal-stress induced genes, including CzrA and ArsR regulons. **Cluster IV** contains envelope stress genes, including YtrA regulon (ABC transporter), induced only by PGRP and vancomycin. **Cluster V** contains several thiol stress-induced oxidoreductases (*trxB, yfmJ, ycnH, yvrD*) and σ^B^-controlled stress response genes. The results for *E. coli* Zn are from NCBI GEO GSE26187, and *E. coli* for H_2_O_2_ and *B. subtilis* for vancomycin, diamide, H_2_O_2_, and Zn are from references 19, 29, 72–74. In some of these studies there was general low induction of gene expression (especially H_2_O_2_ groups).
(TIF)

Figure S4 **Glutathione- and bacillithiol-deficient mutants have reduced total thiols.** WT and glutathione-deficient Δ*gshA E. coli* or bacillithiol-deficient Δ*bshC B. subtilis* mutants were incubated aerobically with albumin (50 µg/ml), or diamide (250 µM), or PGRP (PGLYRP3, 50 µg/ml), and after 30 min intracellular thiols were measured. The results are means ± SEM of 3 experiments (SEM were within symbols, if not visible); the experiment was repeated once with PGLYRP3:PGLYRP4 with similar results (not shown); *, *P*<0.05 WT *vs* Δ*gshA* or Δ*bshC* mutants.
(TIF)

Figure S5 **PGRP induces increase in intracellular Zn in** ***E. coli*** **and** ***B. subtilis*** **and in intracellular Cu in** ***B. subtilis*** **.** *E. coli* or *B. subtilis* were incubated with the indicated compounds without or with Zn^2+^ chelator TPEN (100 µM) as indicated, and assayed for free (labile) intracellular Zn^2+^ concentration with Zinpyr-1 probe (**A**) or for free (labile) intracellular Cu^+^ concentration with CF4 probe (**B**) by flow cytometry, as described in Figure 5A. Representative dot plots are shown; MFI, mean fluorescence intensity; FCSA, forward scatter.
(TIF)

Figure S6 **Δ** ***zntA*** **Δ** ***zitB*** **Zn efflux mutant is highly sensitive to killing by Zn, but Cu efflux mutants have no increased sensitivity to killing by Cu and PGRP.** (**A**) *E. coli* or (**B**) *B. subtilis* (WT or indicated mutants) were incubated aerobically without or with ZnSO_4_ (13 µM) or CuSO_4_ (*E. coli*, 60–75 µM; *B. subtilis*, 300 µM), as indicated, and the numbers of bacteria were determined. (**C**) *E. coli* or *B. subtilis* (WT or indicated mutants) were incubated aerobically with albumin or PGRP (PGLYRP4, *E. coli*, 50 µg/ml; *B. subtilis*, 25 µg/ml) as indicated, and the numbers of bacteria were determined. The results are means ± SEM of 3 experiments (SEM were within symbols, if not visible); *, *P*<0.05; **, *P*<0.001; WT *vs* mutant.
(TIF)

Figure S7 **PGRP equally kills antibiotic-sensitive and antibiotic-resistant bacteria.** WT (antibiotic sensitive) or antibiotic-resistant clinical isolates (*E. coli* NDM-1, resistant to all β-lactams and aminoglycosides, or *S. aureus* MRSA, resistant to penicillins, cephalosporins, macrolides, and aminoglycosides) were incubated aerobically with PGRP (PGLYRP3, 50 µg/ml for *E. coli*; or PGLYRP4, 100 µg/ml for *S. aureus*) or albumin, and the numbers of bacteria were determined. The results are means ± SEM of 3 experiments (SEM were within symbols, if not visible); there were no significant differences between the numbers of surviving antibiotic-sensitive and antibiotic-resistant bacteria.
(TIF)

Table S1 **Top** ***E. coli*** **genes up-regulated by PGRP, gentamicin, and CCCP.**
(PDF)

Table S2 **Top** ***B. subtilis*** **genes up-regulated by PGRP, gentamicin, and CCCP.**
(PDF)

Table S3 **Top** ***E. coli*** **genes down-regulated by PGRP, gentamicin, and CCCP.**
(PDF)

Table S4 **Top** ***B. subtilis*** **genes down-regulated by PGRP, gentamicin, and CCCP.**
(PDF)

Table S5 **Bacterial strains used in this study.**
(PDF)

Table S6 ***E. coli*** **and** ***B. subtilis*** **primers for qRT-PCR.**
(PDF)
