# Supplementary figures and images for: Peptidoglycan Recognition Proteins Kill Bacteria by Inducing Oxidative, Thiol, and Metal Stress
Source: PLoS Pathog. 2014 Jul 17;10(7):e1004280. doi: 10.1371/journal.ppat.1004280 (PMC4102600; doi:10.1371/journal.ppat.1004280)

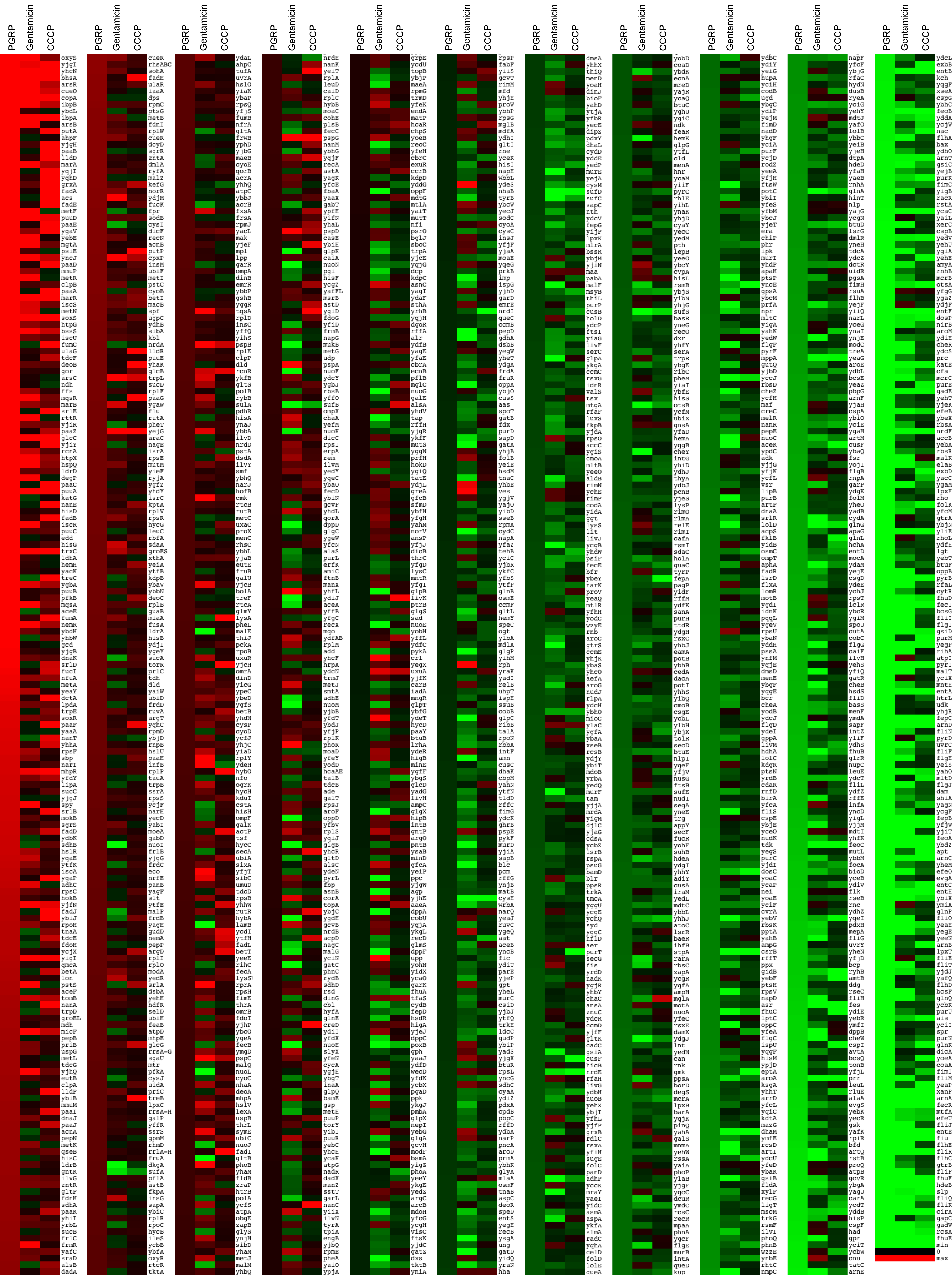

Supplement: Figure S1 — E. coli genes up-regulated or down-regulated more than 3 times by PGRP, gentamicin, or CCCP. The results are heat-maps of mean ratios of the gene expression signals in PGRP-, gentamicin-, or CCCP-treated to control albumin-treated bacteria determined by whole genome expression arrays from 3 experiments (performed as described in Tables S1 and S3), with maximum and minimum signal intensity set at +10 and −10, and arranged from the highest to the lowest fold induction in PGRP-treated group. The mean expression data, the significance of differences, and the gene functions and regulators of top up- and down-regulated genes are shown in Tables S1 and S3 (all data deposited in NCBI GEO under the accession number GSE44211). (TIF) [file ppat.1004280.s001.tif]

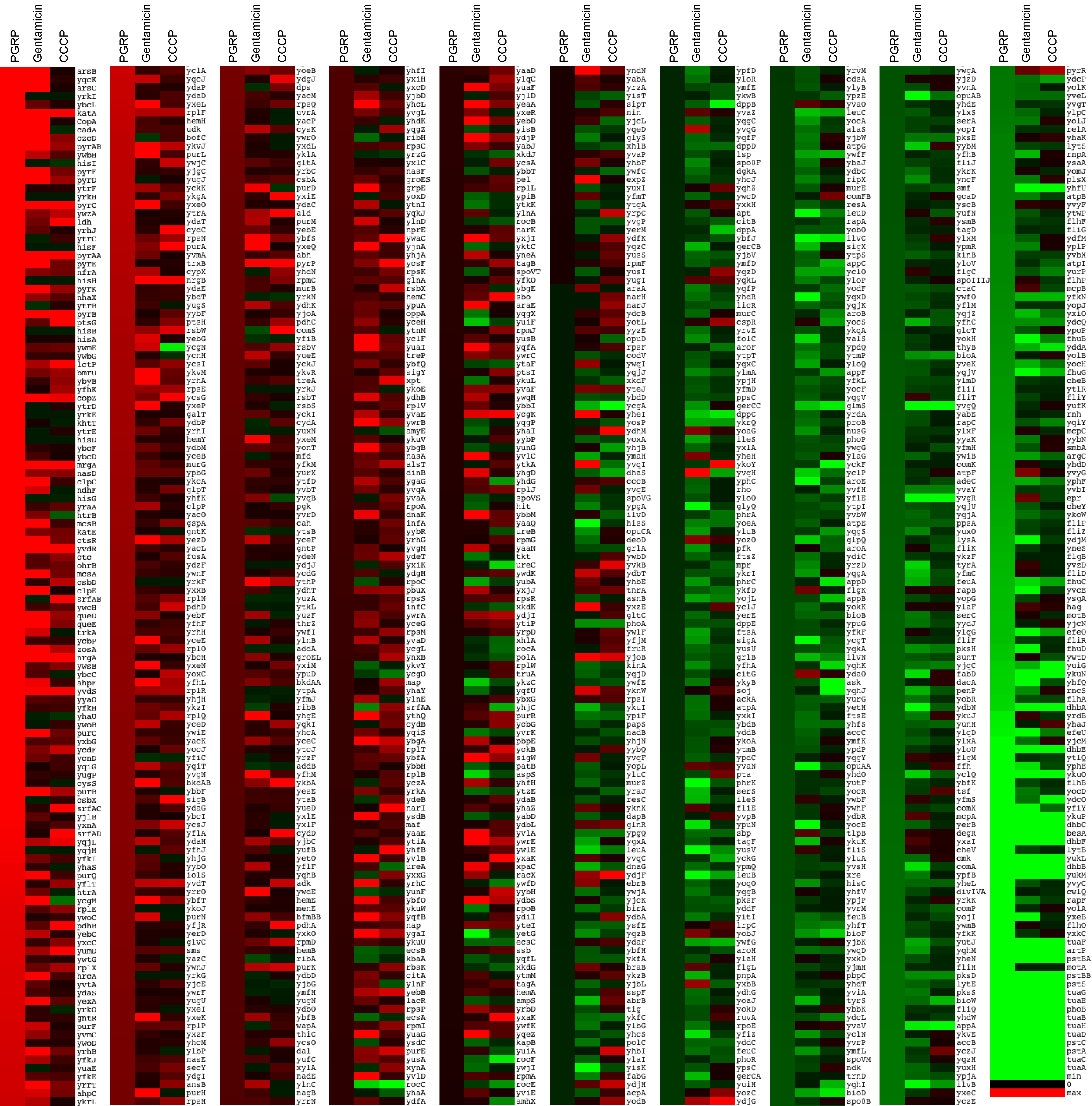

Supplement: Figure S2 — B. subtilis genes up-regulated or down-regulated more than 3 times by PGRP, gentamicin, or CCCP. The results are heat-maps of mean ratios of the gene expression signals in PGRP-, gentamicin-, or CCCP-treated to control albumin-treated bacteria determined by whole genome expression arrays from 3 experiments (performed as described in Tables S2 and S4), with maximum and minimum signal intensity set at +10 and −10, and arranged from the highest to the lowest fold induction in PGRP-treated group. The mean expression data, the significance of differences, and the gene functions and regulators of top up- and down-regulated genes are shown in Tables S2 and S4 (all data deposited in NCBI GEO under the accession number GSE44212). (TIF) [file ppat.1004280.s002.tif]

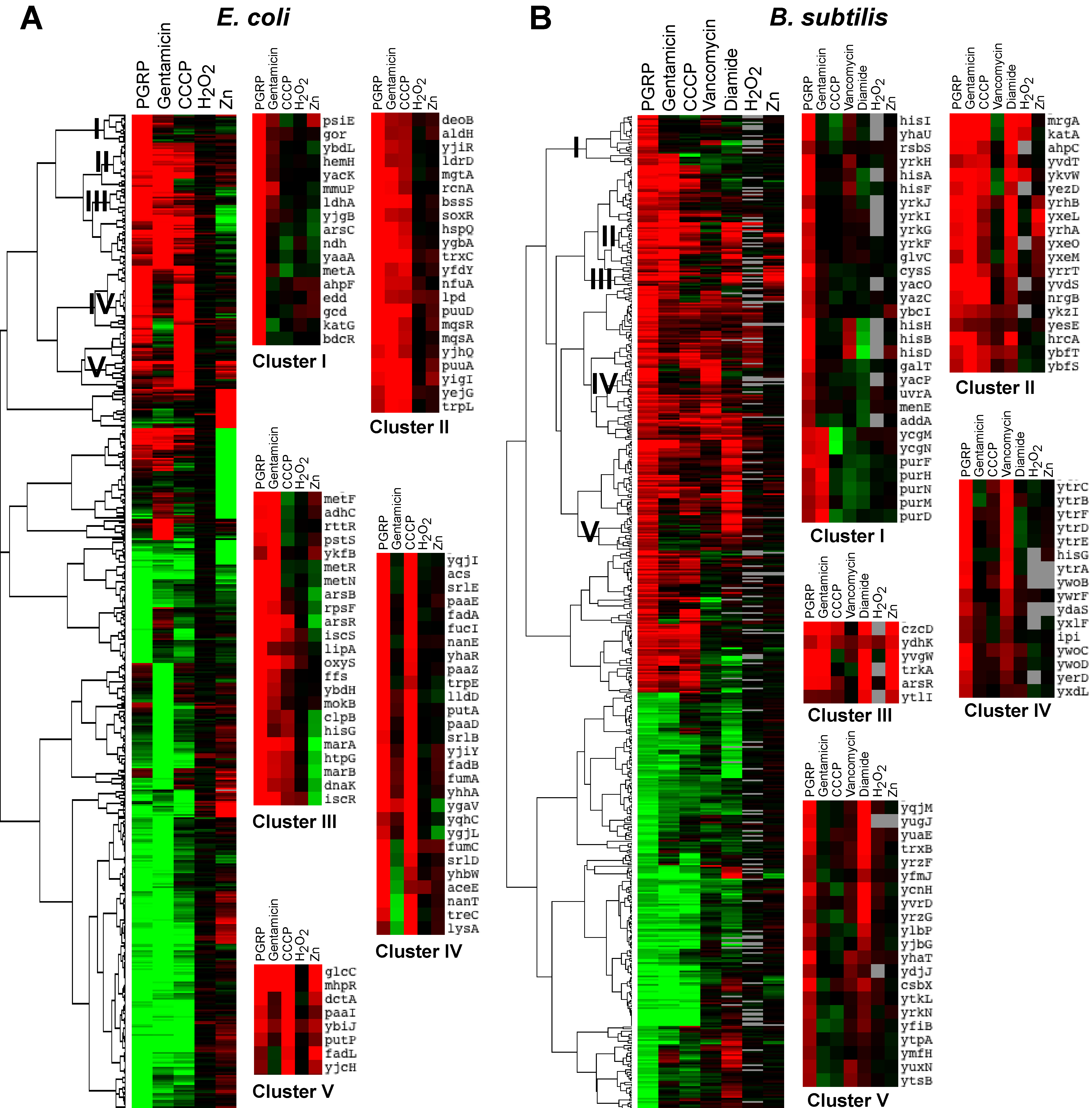

Supplement: Figure S3 — Hierarchical cluster display of 605 ( E. coli ) and 594 ( B. subtilis ) most up-regulated or down-regulated genes in PGRP, gentamicin, CCCP, H2O2, Zn, vancomycin, or diamide treated bacteria. Log2-transfomed gene expression data were clustered based on expression level. (A) E. coli : Cluster I contains genes induced mainly by PGRP (e.g., OxyR-induced: gor, hemH, yaaA, ahpF, katG, and some metal-stress response genes: arsC, ndh). Cluster II contains genes strongly induced by PGRP and also induced by gentamicin and CCCP (e.g., energy acquisition genes and other stress genes). Cluster III contains genes induced by PGRP and gentamicin, including protein and RNA quality control (rttR, clpB, htpG, dnaK), Fe-S cluster repair (iscS, iscR), and some oxidative and metal stress genes (oxyS, arsB, arsR). Cluster IV contains genes induced by PGRP and CCCP (mostly genes for alternative energy sources). Cluster V contains genes induced by PGRP, CCCP, and Zn (genes for several transporters). (B) B. subtilis : Cluster I contains genes induced by PGRP only (e.g., uvrA, addA for DNA repair, and cysS, yrk operons), genes induced by PGRP and vancomycin (his operon), and genes induced by PGRP and gentamicin (ycgM, ycgN for proline utilization, and pur operon for purine synthesis). Cluster II contains genes common to several treatments, including some of the ROS-induced PerR regulon-controlled genes (mrgA, katA, ykvW = zosA) and CymR-rgulated genes for obtaining cysteine and methionine. Cluster III contains several metal-stress induced genes, including CzrA and ArsR regulons. Cluster IV contains envelope stress genes, including YtrA regulon (ABC transporter), induced only by PGRP and vancomycin. Cluster V contains several thiol stress-induced oxidoreductases (trxB, yfmJ, ycnH, yvrD) and σB-controlled stress response genes. The results for E. coli Zn are from NCBI GEO GSE26187, and E. coli for H2O2 and B. subtilis for vancomycin, diamide, H2O2, and Zn are from references 19, 29, 72–74. In [file ppat.1004280.s003.tif]

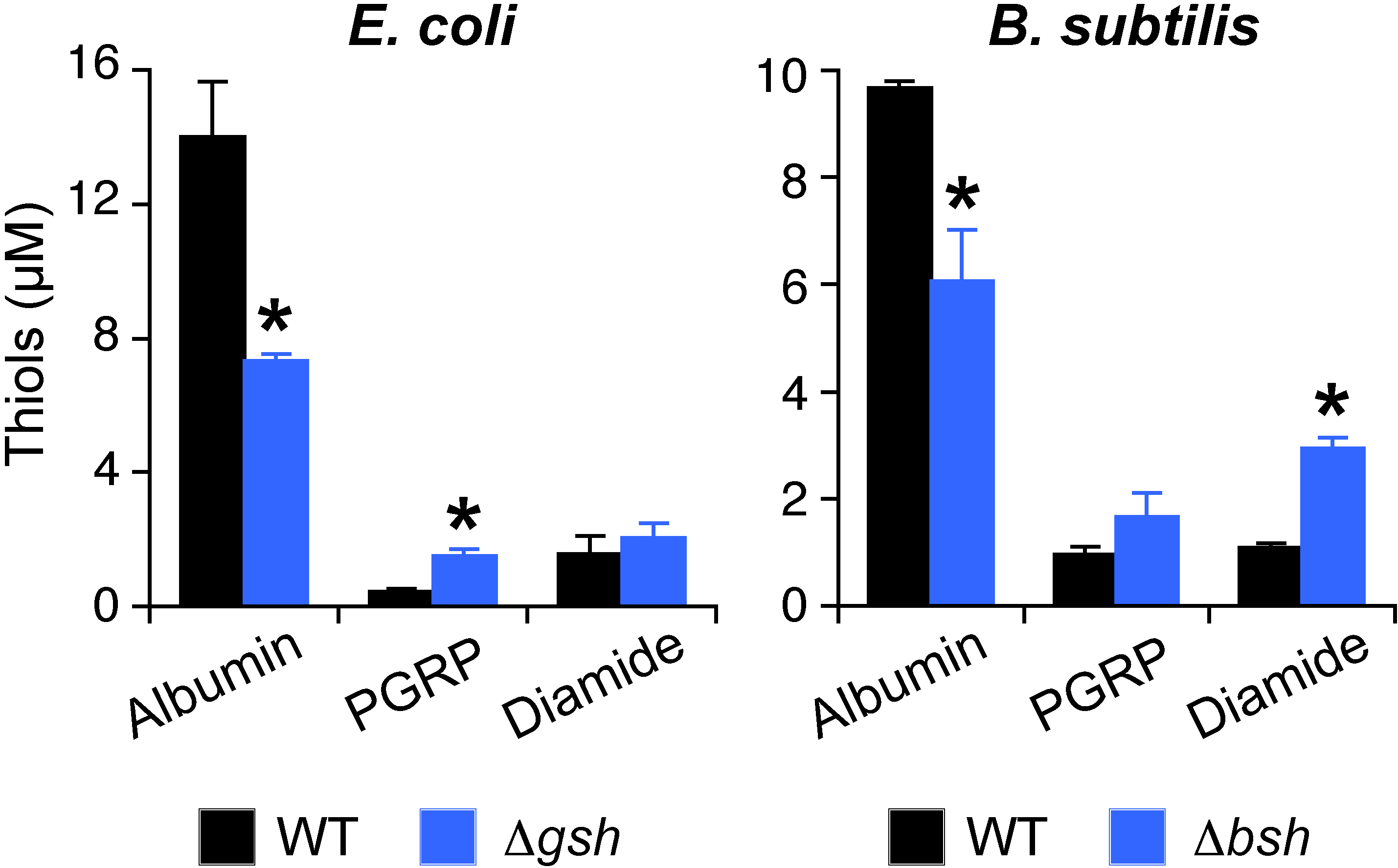

Supplement: Figure S4 — Glutathione- and bacillithiol-deficient mutants have reduced total thiols. WT and glutathione-deficient ΔgshA E. coli or bacillithiol-deficient ΔbshC B. subtilis mutants were incubated aerobically with albumin (50 µg/ml), or diamide (250 µM), or PGRP (PGLYRP3, 50 µg/ml), and after 30 min intracellular thiols were measured. The results are means ± SEM of 3 experiments (SEM were within symbols, if not visible); the experiment was repeated once with PGLYRP3:PGLYRP4 with similar results (not shown); *, P<0.05 WT vs ΔgshA or ΔbshC mutants. (TIF) [file ppat.1004280.s004.tif]

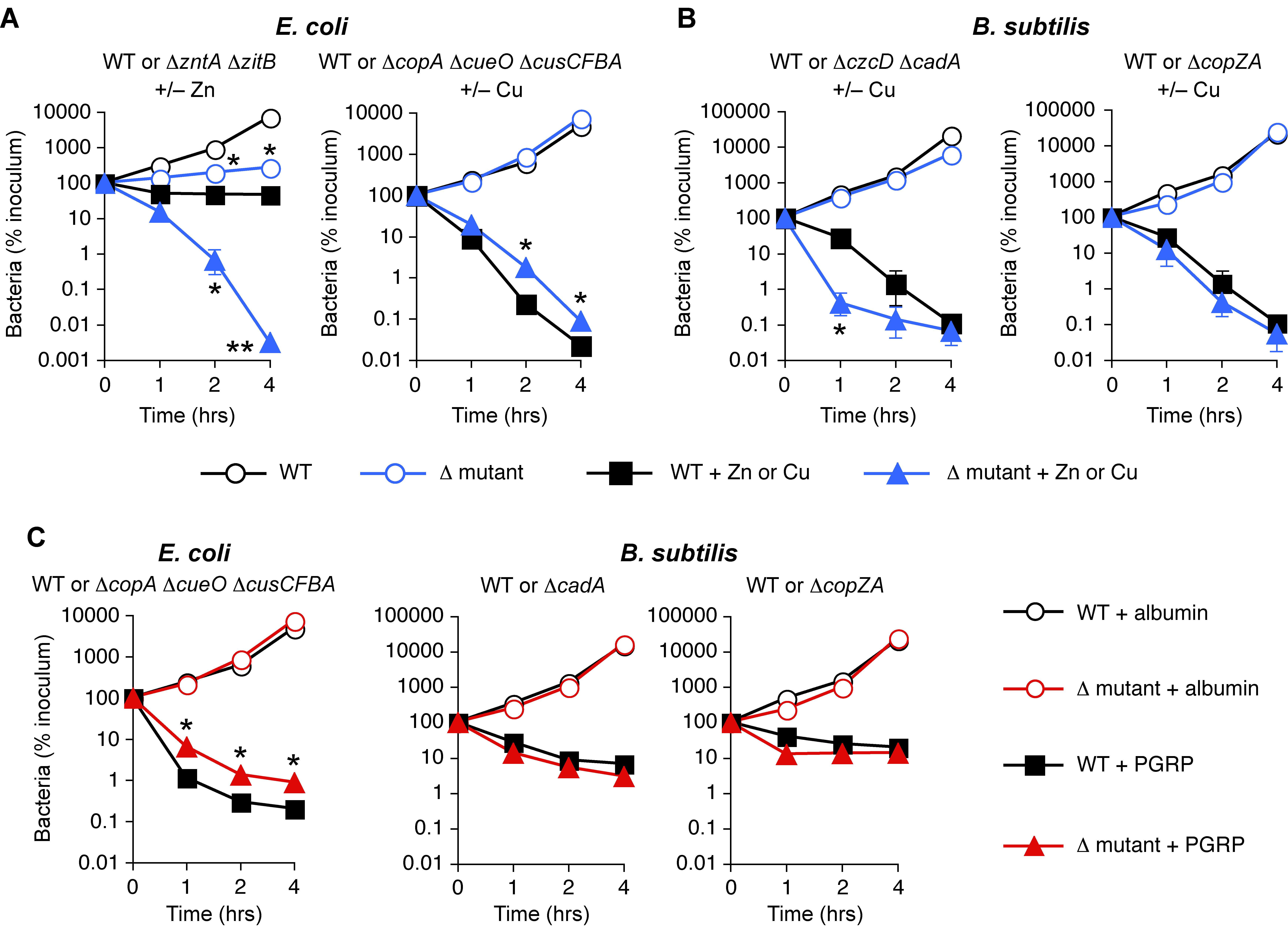

Supplement: Figure S6 — Δ zntA Δ zitB Zn efflux mutant is highly sensitive to killing by Zn, but Cu efflux mutants have no increased sensitivity to killing by Cu and PGRP. (A) E. coli or (B) B. subtilis (WT or indicated mutants) were incubated aerobically without or with ZnSO4 (13 µM) or CuSO4 (E. coli, 60–75 µM; B. subtilis, 300 µM), as indicated, and the numbers of bacteria were determined. (C) E. coli or B. subtilis (WT or indicated mutants) were incubated aerobically with albumin or PGRP (PGLYRP4, E. coli, 50 µg/ml; B. subtilis, 25 µg/ml) as indicated, and the numbers of bacteria were determined. The results are means ± SEM of 3 experiments (SEM were within symbols, if not visible); *, P<0.05; **, P<0.001; WT vs mutant. (TIF) [file ppat.1004280.s006.tif]

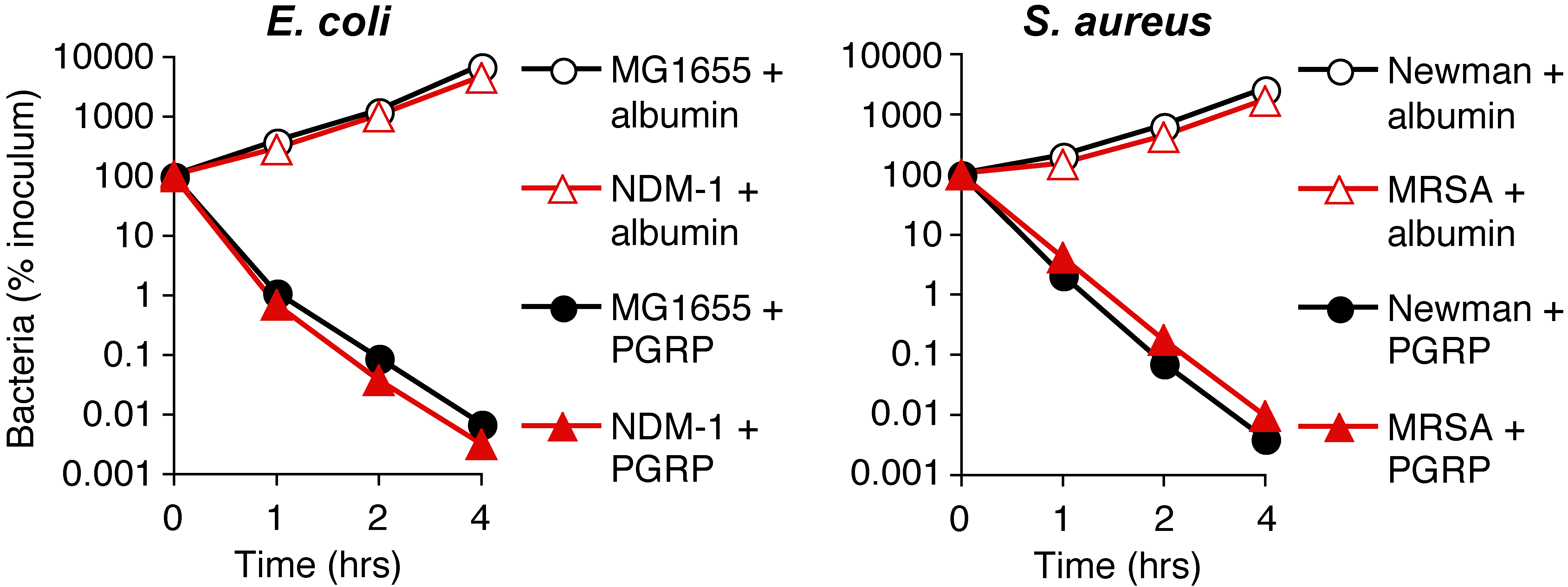

Supplement: Figure S7 — PGRP equally kills antibiotic-sensitive and antibiotic-resistant bacteria. WT (antibiotic sensitive) or antibiotic-resistant clinical isolates (E. coli NDM-1, resistant to all β-lactams and aminoglycosides, or S. aureus MRSA, resistant to penicillins, cephalosporins, macrolides, and aminoglycosides) were incubated aerobically with PGRP (PGLYRP3, 50 µg/ml for E. coli; or PGLYRP4, 100 µg/ml for S. aureus) or albumin, and the numbers of bacteria were determined. The results are means ± SEM of 3 experiments (SEM were within symbols, if not visible); there were no significant differences between the numbers of surviving antibiotic-sensitive and antibiotic-resistant bacteria. (TIF) [file ppat.1004280.s007.tif]
