# Supplementary material for: Peptidoglycan Recognition Proteins Kill Bacteria by Inducing Oxidative, Thiol, and Metal Stress
Source: PLoS Pathog. 2014 Jul 17;10(7):e1004280. doi: 10.1371/journal.ppat.1004280 (PMC4102600; doi:10.1371/journal.ppat.1004280)
Supplement: Table S1 — Top E. coli genes up-regulated by PGRP, gentamicin, and CCCP. (PDF) [file ppat.1004280.s008.pdf]

Table S1. Top *E. coli* genes up-regulated by PGRP, gentamicin, and CCCP [a].

Genes (regulators)	Function	Gene array							qRT PCR				
		PGRP	Gent	CCCP	*P* (*t*-test) [b]		FDR *q* [c]		PGRP	Gent	CCCP	*P* (*t*-test) [b]	
**Oxidative stress**													
oxyS (OxyR, σ^D)	sRNA, detoxification of oxidative damage	711±236	67±35	5±0	**0.0270**	**0.0201**	0.0797	**0.0536**	60±9	3±0.8	1±0.1	**0.0000**	**0.0000**
yjgI	Oxidoreductase/biofilm dispersal mediator	297±36	9±2	73±12	**0.0006**	**0.0020**	**0.0090**	**0.0174**					
yhcN	Stress-induced, Cd and H_2O_2 resistance	276±22	24±0	105±12	**0.0002**	**0.0012**	**0.0052**	**0.0146**	166±48	15±3	145±38	**0.0043**	**0.3473**
ahpF (OxyR, σ^D, σ^E)	Alkyl hydroperoxide reductase, scavenges ROS	53±4	1±0	1±0.1	**0.0001**	**0.0001**	**0.0041**	**0.0068**	12±2	0.3±0	0.7±0.2	**0.0002**	**0.0002**
paaB (Crp, Ihf, σ^D)	Phenylacetyl-CoA oxygenase, detoxifies epoxide and ROS	43±34	1±0	117±74	0.1399	0.2073	0.2629	0.2164					
yqhD	Aldehyde reductase, reduces toxic oxidized glyoxal	35±5	2±1	13±1	**0.0015**	**0.0073**	**0.0134**	**0.0320**					
paaE (Crp, Ihf, σ^D)	NAD(P)H oxidoreductase, detoxifies epoxide and ROS	31±22	1±0	28±10	0.1213	0.4547	0.2356	0.3809					
paaD (Crp, Ihf, σ^D)	Phenylacetyl-CoA epoxidase, detoxifies epoxide and ROS	24±17	1±0	32±12	0.1266	0.3569	0.2432	0.3202					
paaA (Crp, Ihf, σ^D)	Phenylacetyl-CoA epoxidase, detoxifies epoxide and ROS	23±17	1±0	103±65	0.1395	0.1496	0.2623	0.1776					
soxS (SoxR, σ^D)	Superoxide-responsive transcriptional regulator, activates multidrug resistance	20±3	65±5	25±2	**0.0005**	0.1055	**0.0080**	0.1416					
paaZ (Crp, Ihf, σ^D)	Oxepin-CoA hydrolase, detoxifies epoxide and ROS	15±9	1±0	7±4	0.0870	0.2101	0.1840	0.2186					
paaC (Crp, Ihf, σ^D)	Phenylacetyl-CoA epoxidase, detoxifies epoxide and ROS	14±11	1±0	50±31	0.1498	0.1685	0.2775	0.1918					
katG (OxyR, Fnr, σ^D)	Catalase/hydroperoxidase, degrades H_2O_2	13±1	0±0	1±0.2	**0.0004**	**0.0005**	**0.0072**	**0.0111**					
trxC (OxyR, σ^D)	Thioredoxin	12±2	11±1	12±0.5	0.3228	0.3716	0.5008	0.3297					
yhbW (σ^E)	Suppressor of essentiality of rpoE, oxidation/reduction	10±3	0±0	5±2	**0.0144**	0.0844	**0.0515**	0.1245	5±1	0.4±0	1±0.4	**0.0041**	**0.0149**
soxR (σ^D)	Superoxide and redox responsive activator of *soxS*, Fe-S center for redox sensing	9±1	5±0	4±0.8	**0.0095**	**0.0123**	**0.0388**	**0.0408**					
paaF (Crp, Ihf, σ^D)	Dehydroadipyl-CoA hydratase, detoxifies epoxide, ROS	9±5	1±0	15±4	0.0923	0.1939	0.1927	0.2061					
yaaA (OxyR)	Peroxide resistance, lowers intracellular Fe	9±1	1±0	1±0.2	**0.0016**	**0.0013**	**0.0137**	**0.0150**					
**Copper and zinc efflux and detoxification**													
bhsA (Crp, ComR)	Outer membrane protein, Cu permeability	185±26	112±17	201±31	**0.0398**	0.3554	0.1047	0.3191					
cueO (CueR)	Multicopper oxidase, Cu oxidation and detoxification	155±20	16±5	8±2	**0.0012**	**0.0009**	**0.0121**	**0.0135**	56±14	5±0.8	2±0.5	**0.0010**	**0.0007**
copA (CueR, CpxRA)	Cu efflux transporter	121±7	38±18	31±6	**0.0061**	**0.0003**	**0.0300**	**0.0099**	42±12	9±2	9±1	**0.0102**	**0.0097**
grxA (OxyR, σ^D)	Glutaredoxin 1, protects from Co, Cu, Ag, Zn toxicity	35±3	4±1	16±2	**0.0005**	**0.0043**	**0.0078**	**0.0246**	13±2	1±0.2	11±1	**0.0001**	0.1812
gor (OxyR, σ^D)	Glutathione oxidoreductase, Co, Cu, Ag, Zn, As defense	17±2	2±0	2±0.1	**0.0004**	**0.0003**	**0.0071**	**0.0100**					
ndh (ArcAB, Fis)	NADH:ubiquinone oxidoreductase II, cupric reductase	17±1	1±0	0±0.1	**0.0000**	**0.0000**	**0.0025**	**0.0033**	6±1	1±0.5	1±0.2	**0.0018**	**0.0009**

Table S1. Continued

**Arsenite efflux and detoxification**													
arsR (ArsR, σ^D)	Regulator of resistance to As and Sn	176±19	15±1	7±3	**0.0005**	**0.0004**	**0.0080**	**0.0111**	72±15	8±2	4±1	**0.0014**	**0.0010**
arsB (ArsR, σ^D)	As/Sn efflux transporter	62±10	12±2	1±0.1	**0.0036**	**0.0016**	**0.0221**	**0.0158**	39±9	7±1	4±1	**0.0040**	**0.0024**
ygaV	Putative As resistance regulator, resistance to thiol stress	27±6	6±1	81±7	**0.0118**	**0.0022**	**0.0447**	**0.0179**					
arsC (ArsR, σ^D)	Arsenate reductase, reduces arsenate to arsenite	17±3	1±0	1±0.1	**0.0023**	**0.0021**	**0.0172**	**0.0176**	14±2	1±0.1	1±0.2	**0.0004**	**0.0004**
**Metal stress and drug resistance**													
marA (Crp, Fis, SoxR, σ^D)	Transcriptional activator, antibiotic and metal resistance	36±12	17±3	7±1	0.0964	**0.0358**	0.1989	0.0748	26±4	16±6	11±4	0.0573	**0.0074**
yebE (CpxRA, σ^D)	Te resistance terB-like protein	27±3	18±2	1±0.1	**0.0224**	**0.0003**	0.0696	**0.0097**					
mgtA (PhoPQ)	ATP-dependent Mg influx, resistance to Co	26±1	13±1	4±1	**0.0007**	**0.0001**	**0.0095**	**0.0068**					
marR (Crp, Fis, SoxR, σ^D)	MarA/MarB repressor, promotes Co, Ag, Zn, Se toxicity	22±3	18±3	15±3	0.1594	0.0572	0.2905	0.0991					
marB (Crp, Fis, SoxR, σ^D)	Resistance to multiple antibiotics and metals	17±6	6±1	4±0.6	0.0671	**0.0451**	0.1518	0.0857	4±0.7	3±0.9	1±0.2	0.2242	**0.0008**
rcnA (RcnR, Fur, σ^D)	Ni and Co efflux transporter	15±1	8±1	3±0.1	**0.0072**	**0.0003**	**0.0330**	**0.0100**	6±1	1±0.5	1±0.1	**0.0025**	**0.0013**
**Chaperones, protein and RNA quality control, thiol stress**													
ibpB (Ihf, σ^H, σ^N)	Chaperone, refolds misfolded proteins	100±64	281±139	7±2	0.1503	0.1111	0.2780	0.1460	30±5	133±4	2.3±0.3	**0.0000**	**0.0003**
ibpA (Ihf, σ^H, σ^N)	Chaperone, oxidative stress and Cu resistance	79±26	74±30	30±2	0.4547	0.0667	0.6530	0.1085					
yjgH	mRNA endoribonuclease, mRNA quality control	49±10	3±1	17±4	**0.0057**	**0.0213**	**0.0285**	0.0556					
clpB (σ^D, σ^H)	Protein disaggregation, protein folding	23±3	3±1	4±0.5	**0.0021**	**0.0022**	**0.0163**	**0.0177**	14±2	2±1	2±0.6	**0.0007**	**0.0005**
htpG (σ^H)	Chaperon, de novo protein folding	20±4	6±3	4±1	**0.0220**	**0.0097**	0.0688	**0.0363**	7±1	2±1	2±0.6	**0.0104**	**0.0306**
tdcF (Crp, Fnr, Ihf)	mRNA endoribonuclease, RNA quality control	19±10	1±0	7±0.3	0.0708	0.1548	0.1573	0.1809					
ffs (sRNA regulator)	SRP, targeting proteins to membranes	17±13	6±2	2±0.7	0.2228	0.1528	0.3737	0.1797					
rttR (sRNA regulator)	rtT sRNA, RNA quality control	16±8	50±8	1±0	**0.0203**	0.0654	0.0650	0.1073					
yjiR	Predicted aminotransferase, amino acids metabolism	16±4	5±0	4±0.5	**0.0226**	**0.0183**	0.0702	**0.0513**					
htpX (σ^H)	Membrane endopeptidase, protein quality control	15±2	12±1	6±0.6	0.1143	**0.0041**	0.2259	**0.0240**	9±1	15±4	4±0.4	0.0731	**0.0009**
hspQ (σ^H)	Heat shock, excludes denatured proteins	15±2	11±2	5±0.8	0.1549	**0.0088**	0.2843	**0.0348**	7±1	9±1	3±0.5	0.1578	**0.0143**
degP (CpxRA, σ^E)	Serine endoprotease, degrades misfolded proteins	14±3	18±2	1±0.1	0.1962	**0.0043**	0.3380	**0.0246**	7±2	19±2	1±0.2	**0.0024**	**0.0145**
nemR (σ^D)	Repressor of *nemRA-gloA* operon, induced by thiol stress	10±1	12±3	6±1	0.2573	**0.0352**	0.4201	0.0742					
**Methionine and histidine synthesis**													
ybdL	Methionine aminotransferase, Met salvage pathway	94±18	2±0	1±0.2	**0.0037**	**0.0035**	**0.0226**	**0.0222**	33±5	1±0.3	1±0.4	**0.0001**	**0.0001**

Table S1. Continued

metF (σ^D)	Methylenetetrahydrofolate reductase, Met synthesis	31±7	20±3	0±0.1	0.0934	**0.0050**	0.1943	**0.0262**					
mmuP	S-methylmethionine transporter	23±7	6±2	1±0	**0.0295**	**0.0136**	0.0848	**0.0430**	23±6	5±0.7	1±0.1	**0.0105**	**0.0053**
metR	Methionine synthase regulator	23±3	19±3	1±0.1	0.1677	**0.0006**	0.3022	**0.0118**					
metN (YjiE, σ^D)	Methionine transporter ATP-binding subunit	21±2	9±1	1±0.1	**0.0038**	**0.0005**	**0.0228**	**0.0112**	11±2	4±0.6	0.7±0.2	**0.0090**	**0.0010**
hisD (Crp)	Histidinol dehydrogenase, histidine synthesis	13±2	1±0	2±0.4	**0.0014**	**0.0024**	**0.0132**	**0.0181**					
hisG (Crp)	ATP phosphoribosyltransferase, histidine synthesis	12±0	2±1	3±0.2	**0.0000**	**0.0000**	**0.0031**	**0.0033**					
metA (MetR, σ^D, σ^H)	Homoserine transsuccinylase, Met synthesis	9±3	2±0	0±0	**0.0282**	**0.0166**	0.0820	**0.0484**					
**Energy**													
putA (MarA, σ^D)	Proline dehydrogenase, generates glutamate	56±19	2±0	70±27	**0.0219**	0.3505	0.0868	0.3161					
lldD (LldR, σ^D)	Lactate dehydrogenase, aerobic respiration	36±17	2±0	44±20	0.0596	0.3880	0.1401	0.3402					
fadA (Fis, σ^D)	Ketoacyl-CoA thiolase, energy from fatty acids	34±9	1±0	20±6	**0.0109**	0.1231	**0.0426**	0.1563					
acs (Crp, σ^D)	Acetyl-CoA synthetase, TCA cycle	33±7	1±0	21±8	**0.0047**	0.1465	**0.0254**	0.1754					
fadE (σ^D)	Acyl-CoA dehydrogenase, energy from fatty acids	32±7	2±0	11±2	**0.0070**	**0.0249**	**0.0327**	0.0615					
puuD	Glutamyl-GABA hydrolase, energy from putrescine, induced in stress	31±3	45±2	9±1	**0.0093**	**0.0013**	**0.0386**	**0.0150**					
fumC (MarA, SoxR, σ^S)	Fumarate hydratase, TCA cycle	19±3	0±0	11±1	**0.0022**	**0.0460**	**0.0168**	0.0865	8±1	1±0.5	6±2	**0.0010**	0.1578
ulaG	Ascorbate-phosphate lactonase, energy from ascorbate by fermentation	19±2	2±0	4±0.5	**0.0006**	**0.0011**	**0.0089**	**0.0139**					
srlE (Crp, GutM, Hns, σ^D)	Sorbitol PTS permease, alternative energy	16±7	1±0	13±6	**0.0438**	0.3724	0.1124	0.3300					
glcC (Crp, σ^D)	Activator for *glc* operon, energy from glyoxylate	15±2	13±1	12±2	0.1126	0.1483	0.2233	0.1767					
yjiY	Putative carbon starvation protein	15±6	2±0.4	38±24	**0.0455**	0.2056	0.1153	0.2153					
puuA	Glutamate-putrescine ligase, energy from putrescine	14±3	30±4	16±2	**0.0143**	0.2477	**0.0512**	0.2462					
nanE (Crp, σ^D)	N-acetylmannosamine-6-P epimerase	13±2	1±0	10±5	**0.0020**	0.3006	**0.0159**	0.2835					
fadB (Fis, σ^D)	Hydroxybutyryl-CoA epimerase, using fatty acids	13±5	2±0	18±4	**0.0442**	0.2235	0.1128	0.2284					
puuC	Glu-aminobutyraldehyde dehydrogenase, energy from putrescine	12±2	3±0	4±1	**0.0086**	**0.0142**	**0.0367**	**0.0441**					
edd (σ^D)	Phosphogluconate dehydratase, using gluconate	12±4	1±0	1±0.1	**0.0206**	**0.0216**	0.0658	0.0560					
ldhA (σ^H)	D-lactate dehydrogenase, fermentation	12±1	2±1	1±0.1	**0.0003**	**0.0000**	**0.0066**	**0.0033**					
treC (ArcA, Crp, σ^D)	Trehalose hydrolase, energy from trehalose	11±3	0.3±0	26±6	**0.0079**	**0.0406**	**0.0347**	0.0810					
puuB	Glutamylputrescine oxidase, energy from putrescine	11±2	2±1	2±1	**0.0036**	**0.0063**	**0.0223**	**0.0291**					
pfkB (σ^S)	Phosphofructokinase, energy from glycolysis	11±1	2±0	4±0.5	**0.0001**	**0.0007**	**0.0046**	**0.0128**					
aceE (Crp, Fnr, σ^D)	Pyruvate dehydrogenase, TCA cycle	11±3	0±0	3±0.4	**0.0183**	**0.0435**	0.0605	0.0841					
fumA (Crp, σ^D)	Fumarate hydratase, TCA cycle	11±0	2±0	11±1	**0.0000**	0.4902	**0.0030**	0.3993					

Table S1. Continued

ybdH	Predicted oxidoreductase	10±1	4±1	2±0.2	**0.0127** **0.0011**	**0.0471** **0.0140**				
gcd (Ihf, σ^D)	Glucose dehydrogenase, Entner-Doudoroff pathway	10±1	1±0	1±0.1	**0.0010** **0.0010**	**0.0113** **0.0136**				
fucI (Crp, FucR, σ^D)	L-fucose isomerase, energy from fucose	10±1	1±0	8±1	**0.0002** 0.1431	**0.0055** 0.1728				
**Fe-S clusters**										
yqjI (Fnr)	Repressor of Fe reductase *yqjH*	35±7	1±0	34±4	**0.0036** 0.4345	**0.0221** 0.3685				
iscS (IscR, σ^D)	Cysteine desulfurase, Fe-S cluster repair	22±0	9±1	4±0.1	**0.0001** **0.0000**	**0.0032** **0.0009**				
iscU (IscR, σ^D)	Fe-S cluster assembly scaffold protein	19±1	5±0	4±0.2	**0.0001** **0.0001**	**0.0038** **0.0057**	19±7	3±0.8	3±0.6	**0.0169** **0.0169**
iscR (σ^D)	Regulator of Fe-S cluster assembly genes	13±1	11±1	3±0.2	0.0965 **0.0003**	0.1990 **0.0095**				
hemH (OxyR, σ^D)	Ferrochelatase, Fe acquisition	11±1	2±0	1±0.1	**0.0009** **0.0006**	**0.0107** **0.0017**				
nfuA (Crp, σ^D, σ^H)	Non-ISC, non-SUF Fe-S synthesis factor	10±1	6±1	6±0.2	**0.0334** **0.0124**	0.0927 **0.0410**				
**DNA repair**										
deoB (Crp, Fis, σ^D)	Phosphopentomutase, nucleosides and nucleotides salvage	19±4	4±0	3±0.6	**0.0097** **0.0091**	**0.0391** **0.0352**				

[a] Bacteria were treated with albumin (100 μg/ml, control), PGRP (PGLYRP4, 100 μg/ml), or gentamicin (5 μg/ml), for 30 min or CCCP (800 μM) for 15 min at 37°C, and gene expression was determined by whole genome expression arrays and qRT-PCR. Genes are listed from the most to the least up-regulated in each group. The numbers are mean ratios ± SEM of the gene expression signals in PGRP-, gentamicin-, or CCCP-treated bacteria to control albumin-treated bacteria, obtained from 3 independent whole genome expression arrays experiments and 3 independent qRT-PCR experiments (as indicated). All genes in PGRP-treated bacteria were expressed significantly higher than in control bacteria at $P<0.001$ by two sample one-tailed *t*-test (not shown), and 95% of these genes were expressed significantly higher than in control at FDR $q\leq0.05$ (not shown). The entire whole genome expression array data have been deposited in NCBI GEO under the accession number GSE44211.

[b] *P* values (two sample one-tailed *t*-test) for the differences in gene expression in PGRP-treated bacteria *versus* gentamicin- or CCCP-treated bacteria (numbers on the left or on the right, respectively) are shown as indicated, with $P\leq0.05$ in bold.

[c] FDR (false discovery rate) *q* values for the differences in gene expression in PGRP-treated bacteria *versus* gentamicin- or CCCP-treated bacteria (numbers on the left or on the right, respectively) are shown as indicated, with $q\leq0.05$ in bold.
