# Supplementary material for: Peptidoglycan Recognition Proteins Kill Bacteria by Inducing Oxidative, Thiol, and Metal Stress
Source: PLoS Pathog. 2014 Jul 17;10(7):e1004280. doi: 10.1371/journal.ppat.1004280 (PMC4102600; doi:10.1371/journal.ppat.1004280)
Supplement: Table S2 — Top B. subtilis genes up-regulated by PGRP, gentamicin, and CCCP. (PDF) [file ppat.1004280.s009.pdf]

Table S2. Top *B. subtilis* genes up-regulated by PGRP, gentamicin, and CCCP [a].

Genes (regulators)	Function	Gene array					qRT PCR			
		PGRP	Gent	CCCP	*P* (*t*-test) [b]	FDR *q* [c]	PGRP	Gent	CCCP	*P* (*t*-test) [b]
**Oxidative stress**										
yrkl	Predicted NAD(P)H oxidoreductase	60±30	1±0	1±0	0.0625 0.0625	0.0824 0.0815				
katA (PerR)	Vegetative catalase 1, degrades H_2O_2	54±24	94±26	13±3	0.1623 0.0822	0.1238 0.0908	417±45	469±97	48±6	0.3239 **0.0001**
nfrA (Spx, σ^A, σ^D)	NADPH nitro/flavin reductase, response to thiol stress and oxidative stress	28±1	5±2	4±0	**0.0003** **0.0000**	**0.0093** **0.0039**	14±3	3±1	2±0.5	**0.0066** **0.0040**
ybcF	Carbonic anhydrase-like protein, maintains pH	17±8	2±0	1±0	0.0642 0.0586	0.0834 0.0804	191±49	17±4	8±0.4	**0.0061** **0.0048**
ybcD	Putative aldehyde dehydrogenase, aldehyde oxidation	17±7	3±1	1±0	**0.0472** **0.0346**	0.0743 0.0655	26±5	4±0.5	1±0.2	**0.0031** **0.0017**
mrgA (PerR, σ^A)	Mini-ferritin, protects from oxidative stress	17±6	68±21	22±5	**0.0416** 0.2574	0.0701 0.1537				
ndhF	NADH dehydrogenase, respiration	16±6	8±6	1±0	0.1996 **0.0348**	0.1369 0.0655				
katE (σ^B)	Catalase 2, degrades H_2O_2	14±4	5±2	5±2	0.0620 **0.0511**	0.0823 0.0765	22±6	3±1	2±0.2	**0.0084** **0.0060**
ohrB (σ^B)	Organic hydroperoxide reductase	13±3	3±1	4±1	**0.0244** **0.0352**	0.0567 0.0656				
srfAB (PerR, ComAP)	Surfactin synthetase, thiol stress response	12±9	1±0	15±7	0.1396 0.4004	0.1149 0.1980				
ywcH (σ^D, σ^A)	Putative monooxygenase, detoxification	12±0	3±1	4±1	**0.0001** **0.0001**	**0.0073** **0.0065**	20±4	2±0.4	2±0.3	**0.0015** **0.0014**
ycbP (σ^B)	Putative NAD(P)H-dependent FMN reductase	12±3	2±1	2±0	**0.0181** **0.0144**	**0.0497** **0.0477**				
ahpF (PerR, σ^A)	Alkyl hydroperoxide reductase, degrades H_2O_2	11±4	9±2	2±0	0.3578 0.0560	0.1982 0.0793	36±10	14±4	2±0.5	**0.0484** **0.0087**
yxnA (σ^B)	Oxidoreductase, induced in oxidative stress	10±2	2±0	3±1	**0.0073** **0.0157**	**0.0326** **0.0495**	13±4	2±0.5	2±0.6	**0.0092** **0.0109**
yqjM (σ^A)	Flavin oxidoreductase, reduction of aldehydes and ketones, induced by H_2O_2	10±3	1±0	1±0	**0.0160** **0.0166**	**0.0464** **0.0503**	18±2	1±0.2	1±0.4	**0.0000** **0.0000**
yfkI (σ^B)	Stress protein, protects from H_2O_2	10±6	5±1	3±1	0.2176 0.1478	0.1439 0.1173				
**Copper and zinc efflux and detoxification**										
copA (CsoR, σ^A)	Cu efflux transporting ATPase	53±11	69±5	1±0	0.1193 **0.0045**	0.1060 **0.0306**	93±20	136±15	3±0.4	0.0692 **0.0021**
cadA (CzrA, σ^A)	Cd, Zn, Cu, Co, Ni efflux ATPase	52±12	77±7	1±0	0.0796 **0.0061**	0.0907 **0.0336**	33±9	49±9	1±0.1	0.1711 **0.0187**
czcD (CzrA, σ^A)	K/H- Zn, Cu, Co, Ni efflux antiporter	52±17	49±11	5±2	0.4329 **0.0268**	0.2231 0.0594	34±4	21±6	3±0.8	0.0581 **0.0002**
copZ (CsoR, σ^A)	Cu chaperone for Cu efflux	21±3	30±2	9±3	**0.0288** **0.0218**	0.0611 **0.0548**				
trkA (CzrA, σ^A)	Cation exporter, resistance to metal ions	12±1	8±2	1±0	0.0939 **0.0001**	0.0962 **0.0056**				
**Arsenite efflux and detoxification**										
arsB (ArsR, σ^F, σ^A)	Arsenite efflux pump	151±64	96±33	1±0	0.2448 **0.0398**	0.1548 0.0685	238±33	73±20	2±0.6	**0.0025** **0.0002**
yqcK (ArsR, σ^F, σ^A)	Arsenite efflux pump operon	101±40	71±25	2±0	0.2782 **0.0326**	0.1686 0.0635				
arsC (ArsR, σ^F, σ^A)	Arsenate reductase, reduces arsenate to arsenite	90±39	70±23	1±0	0.3422 **0.0435**	0.1927 0.0712	220±49	167±34	2±0.2	0.2087 **0.0023**
**Other transporters**										
ybcL	MFS-type transporter, unknown function	56±36	4±0	1±0	0.1129 0.1037	0.1036 0.0995				
ytrF (YtrA, σ^A)	ABC transporter, unknown function	37±4	2±0	2±0	**0.0004** **0.0004**	**0.0102** **0.0131**	45±10	2±0.8	2±0.5	**0.0030** **0.0031**
ytrC (YtrA, σ^A)	ABC transporter, unknown function	32±11	1±0	2±1	**0.0220** **0.0259**	**0.0544** 0.0585	62±17	3±0.8	2±0.4	**0.0072** **0.0071**
ytrB (YtrA, σ^A)	ABC transporter, unknown function	25±10	1±0	2±0	**0.0328** **0.0370**	0.0642 0.0671	37±9	1±0.2	2±0.5	**0.0045** **0.0055**
lctP (Rex, σ^A)	L-lactate permease	23±9	7±4	27±8	0.0970 0.3606	0.0977 0.1858				
ytrD (YtrA, σ^A)	ABC transporter, unknown function	21±7	1+0	2±0	**0.0212** **0.0231**	**0.0532** 0.0559	13±1	1±0.2	2±0.3	**0.0001** **0.0001**
khtT	K/H K^+ efflux antiporter, acid stress response	20±2	1±0	1±0	**0.0006** **0.0006**	**0.0119** **0.0153**				
ytrE (YtrA, σ^A)	ABC transporter, unknown function	19±8	1±0	1±0	**0.0428** **0.0459**	0.0711 0.0727	19±4	1±0.3	2±0.3	**0.0013** **0.0016**
yvdR	Multidrug efflux transporter homolog	13±6	18±6	14±5	0.3144 0.4856	0.1826 0.2219				
yvdS	Multidrug efflux transporter homolog	11±5	18±7	12±6	0.2326 0.4628	0.1497 0.2156				
zosA (PerR, σ^A)	Zn uptake ATPase, oxidative/thiol stress	11±5	24±6	7±5	0.0821 0.2893	0.0920 0.1634				

Table S2. Continued

nrgA (TnrA, σ^A)	Ammonium uptake, acid stress response	11±2	36±13	41±23	0.0746	0.1356	0.0881	0.1115					
yhaU	K/H K^+ efflux antiporter, acid stress response	11±1	1±0	1±0	**0.0004**	**0.0004**	**0.0130**	**0.0131**					
**Chaperones, protein quality control, thiol stress**													
nhaX (σ^B)	Stress protein, protects from oxidative damage	26±2	6±2	3±1	**0.0016**	**0.0004**	**0.0172**	**0.0131**	15±1	2±0	2±1	**0.0000**	**0.0001**
clpC (CtsR, σ^B, σ^F, σ^A)	Inducer of autolysins (LytC and LytD)	17±4	10±2	2±1	0.1133	0.0092	0.1073	**0.0393**					
yraA (AdhR, σ^B, σ^M)	Cysteine proteinase, degrades thiol-damaged proteins	15±3	2±1	4±0	**0.0063**	**0.0087**	**0.0308**	**0.0388**	23±3	2±0	1±0.3	**0.0004**	**0.0003**
htrB (CssRS)	HtrA-like serine protease	14±4	5±0	0±0	**0.0390**	**0.0123**	0.0686	**0.0456**	100±33	19±9	2±0.2	**0.0269**	**0.0118**
mcsB (CtsR, σ^B, σ^F)	Arginine kinase, protein degradation, thiol stress	14±4	14±3	3±1	0.4891	**0.0198**	0.2380	**0.0534**					
ctsR (CtsR, σ^B)	Master regulator of protein quality control	14±6	13±6	5±2	0.4612	0.1210	0.2310	0.1061					
ctc (σ^B)	50S ribosomal protein, protein synthesis	13±1	3±1	5±1	**0.0032**	**0.0054**	**0.0225**	**0.0317**					
mcsA (CtsR, σ^B, σ^F)	Activator of protein degradation, thiol stress	13±5	14±6	5±1	0.4548	0.0735	0.2293	0.0860					
clpE (CtsR, σ^A)	Protease chaperone, protein degradation	12±3	113±25	1±0	**0.0077**	**0.0130**	**0.0332**	**0.0465**					
queD (preQ1)	Protein synthesis (trRNA modification)	12±4	23±3	6±1	**0.0541**	0.1389	0.0792	0.1129					
queE (preQ1)	Protein synthesis (trRNA modification)	12±5	25±5	5±1	0.0754	0.1312	0.0884	0.1096					
yyaO	Carboxypeptidase homolog	11±3	3±1	3±0	**0.0338**	**0.0282**	0.0651	0.0597					
yfkH (σ^B)	Putative tRNA-processing ribonuclease	11±6	2±0	2±1	0.1026	0.1095	0.0998	0.1018					
yugP	Putative metal-dependent protease	10±1	2±0	2±0	**0.0002**	**0.0002**	**0.0083**	**0.0100**					
cysS (T-box, σ^A)	Cysteine-tRNA synthetase, protein synthesis	10±3	2±1	1±0	**0.0312**	**0.0246**	0.0630	0.0573					
htrA (CssRS, σ^A)	Serine protease Do (heat shock protein)	9±3	4±0	1±0	0.1023	**0.0297**	0.0997	0.0607	81±21	15±5	1±0.2	**0.0103**	**0.0041**
**Histidine synthesis**													
hisI	Histidine synthesis	44±22	1±0	1±0	0.0582	0.0571	0.0811	0.0797	71±22	1±0.7	0.2±0	**0.0092**	**0.0086**
hisF	Histidine synthesis	31±17	1±0	1±0	0.0736	0.0735	0.0878	0.0860	96±15	2±1	0.5±0	**0.0004**	**0.0003**
hisH	Histidine synthesis	26±15	1±0	1±0	0.0891	0.0887	0.0951	0.0936	108±21	1±0.4	0.1±0	**0.0012**	**0.0011**
hisB	Histidine synthesis	24±14	1±0	1±0	0.0902	0.0902	0.0951	0.0936					
hisA	Histidine synthesis	24±14	1±0	1±0	0.0892	0.0894	0.0951	0.0936					
hisD	Histidine synthesis	18±10	1±0	1±0	0.0885	0.0885	0.0948	0.0935					
hisG	Histidine synthesis	16±10	1±0	1±0	0.0998	0.0998	0.0988	0.0976					
**Energy**													
yrhJ (FatR, σ^M, σ^W, σ^X)	Predicted NADPH-cytochrome reductase, fatty acids	34±8	4±1	1±0	**0.0087**	**0.0067**	**0.0339**	**0.0352**	46±11	19±5	8±2	**0.0326**	**0.0069**
ldh (Rex, σ^A)	Lactate dehydrogenase, respiration, fermentation	34±20	4±3	46±18	0.1123	0.3446	0.1034	0.1809	33±11	6±2	94±20	**0.0226**	**0.0172**
ptsG (GlcT, σ^A)	Glucose transport and phosphorylation	24±17	4±1	16±1	0.1492	0.3244	0.1183	0.1143					
nasD (ResD, TnrA, σ^A)	Nitrite reductase (nitrite as nitrogen source)	17±8	6±2	6±0	0.1301	0.1241	0.1098	0.1073					
ycdF (σ^B)	Predicted glucose dehydrogenase, generates NADPH	11±2	5±1	8±4	**0.0326**	0.2646	0.0640	0.1557					
ycnD	NADPH-FMN oxidoreductase, electron transport	11±4	4±1	3±1	0.1117	0.0690	0.1034	0.0841					
yqiG	Predicted flavin oxidoreductase, electron transport	10±3	1±0	2±1	**0.0186**	**0.0301**	**0.0500**	0.0609					
pdhB (stringent resp., σ^A)	Pyruvate dehydrogenase, TCA cycle	9±5	4±1	18±2	0.1915	0.0967	0.1335	0.0957					
gntR (AbrB, σ^A)	Repressor of gluconate operon	9±3	2±0	2±1	0.0553	0.0579	0.0799	0.0803					
**Envelope lysis and**													

Table S2. Continued

**remodeling**										
ywbH	Putative holin, regulates murein hydrolases	46±15	7±1	3±0	**0.0303** **0.0226**	0.0624 0.0555				
yrkH	Putative hydrolase	34±17	1±0	1±0	0.0636 0.0636	0.0833 0.0820				
ywbG	Putative peptidoglycan hydrolase	23±8	2±0	2±1	**0.0296** **0.0292**	0.0618 0.0604				
ywoC (YtrA, σ^A)	Putative hydrolase	9±1	1±0	2±0	**0.0002** **0.0005**	**0.0091** **0.0148**				
**DNA repair**										
pyrAB (PyrR, σ^A)	Carbamoyl-phosphate synthetase, pyrimidine synthesis	51±26	46±7	6±3	0.3897 **0.0214**	0.2226 0.0915				
pyrF (PyrR, σ^A)	Orotidine-phosphate decarboxylase, pyrimidine synthesis	40±20	45±2	5±2	0.4018 0.0740	0.2138 0.0860				
pyrD (PyrR, σ^A)	Dihydroorotic acid dehydrogenase, pyrimidine synthesis	37±20	38±1	6±3	0.4766 0.1022	0.2346 0.0989				
pyrC (PyrR, σ^A)	Dihydroorotase, pyrimidine synthesis	34±24	59±21	20±3	0.2363 0.2892	0.1513 0.1634				
pyrAA (PyrR, σ^A)	Carbamoyl-phosphate synthetase, pyrimidine synthesis	31±17	84±27	14±2	0.0850 0.1924	0.0933 0.1249				
pyrE (PyrR, σ^A)	Orotate phosphoribosyltransferase, pyrimidine synthesis	29±7	63±4	4±1	**0.0065** **0.0136**	**0.0309** **0.0470**				
pyrK (PyrR, σ^A)	Dihydroorotic acid dehydrogenase, pyrimidine synthesis	26±13	45±1	6±3	0.1055 0.0961	0.1010 0.0956				
pyrB (PyrR, σ^A)	Aspartate carbamoyltransferase, pyrimidine synthesis	25±18	77±22	35±4	0.0680 0.3058	0.0856 0.1693				
purC (PurR)	phosphoribosylaminoimidazole succinocarboxamide synthase, purine synthesis	11±3	36±12	4±1	0.0599 0.0592	0.0813 0.0806				
purB (PurR)	adenylsuccinate lyase, purine synthesis	10±2	27±6	7±2	0.**0309** 0.1761	0.0628 0.1262				
**Detoxification and stress-response**										
ywzA (σ^B)	General stress protein	34±8	5±2	7±3	**0.0118** **0.0164**	**0.0394** **0.0500**				
ywmE (σ^B)	Ethanol stress and general stress	24±6	4±1	4±1	**0.0199** **0.0205**	**0.0520** **0.0538**				
bmrU (σ^B)	Multidrug resistance protein	23±7	5±2	6±2	**0.0337** **0.0422**	0.0651 0.0703				
ybyB (σ^B)	Survival of ethanol stress	22±9	2±1	6±3	**0.0452** 0.0802	0.0729 0.0898				
yfhK (σ^B)	General stress protein, ethanol stress	21±8	1±0	6±3	**0.0335** 0.0795	0.0651 0.0867				
csbD (σ^B)	Survival of salt and temperature stress	12±5	2±1	7±3	**0.0513** 0.2261	0.0777 0.1412				
ywsB (σ^B)	Survival of ethanol and salt stress	11±1	4±1	5±2	**0.0027** **0.0139**	**0.0215** **0.0470**				
yxbG (σ^B)	General stress response	11±3	3±1	4±1	**0.0254** **0.0336**	0.0575 0.0645				
yflT (σ^B)	Ethanol and heat stress	9±2	2±1	6±3	**0.0165** 0.1957	**0.0496** 0.1306				

[a] Bacteria were treated with albumin (100 µg/ml, control), PGRP (PGLYRP4, 100 µg/ml), or gentamicin (5 µg/ml), for 30 min or CCCP (800 µM) for 15 min at 37°C, and gene expression was determined by whole genome expression arrays and qRT-PCR. Genes are listed from the most to the least up-regulated in each group. The numbers are mean ratios ± SEM of the gene expression signals in PGRP-, gentamicin-, or CCCP-treated bacteria to control albumin-treated bacteria, obtained from 3 independent whole genome expression arrays experiments and 3 independent qRT-PCR experiments (as indicated). All genes in PGRP-treated bacteria were expressed significantly higher than in control bacteria at $P<0.001$ by two sample one-tailed *t*-test (not shown), and 94% of these genes were expressed significantly higher than in control at FDR $q\leq0.05$ (not shown). The entire whole genome expression array data have been deposited in NCBI GEO under the accession number GSE44212.

[b] *P* values (two sample one-tailed *t*-test) for the differences in gene expression in PGRP-treated bacteria *versus* gentamicin- or CCCP-treated bacteria (numbers on the left or on the right, respectively) are shown as indicated, with $P\leq0.05$ in bold.

[c] FDR (false discovery rate) *q* values for the differences in gene expression in PGRP-treated bacteria *versus* gentamicin- or CCCP-treated bacteria (numbers on the left or on the right, respectively) are shown as indicated, with $q\leq0.05$ in bold.
