# Supplementary material for: Peptidoglycan Recognition Proteins Kill Bacteria by Inducing Oxidative, Thiol, and Metal Stress
Source: PLoS Pathog. 2014 Jul 17;10(7):e1004280. doi: 10.1371/journal.ppat.1004280 (PMC4102600; doi:10.1371/journal.ppat.1004280)
Supplement: Table S3 — Top E. coli genes down-regulated by PGRP, gentamicin, and CCCP. (PDF) [file ppat.1004280.s010.pdf]

Table S3. Top *E. coli* genes down-regulated by PGRP, gentamicin, and CCCP [a].

Gene (regulators)	Function	PGRP	Gentamicin	CCCP	*P* (*t*-test) [b]	FDR *q* [c]
**Iron uptake**						
fhuE (Fur-, σ^D)	Ferric-rhodotorulic acid transporter	0.019±0.003	0.029±0.007	0.022±0.004	0.1214 0.3246	0.2357 0.2998
cirA (Crp, Fur-, σ^D)	Ferric iron-catecholate transporter	0.024±0.004	0.049±0.015	0.024±0.004	0.0907 0.4851	0.1901 0.3969
fiu (Crp, Fur-, σ^D)	TonB-dependent ferric iron transporter	0.035±0.000	0.035±0.000	0.045±0.010	1.0000 0.1870	0.7000 0.2011
entE (Crp, Fur-, σ^D)	Fe siderophore enterobactin synthesis	0.035±0.006	0.025±0.000	0.038±0.014	0.0818 0.4270	0.1759 0.3638
efeU (σ^D)	Ferrous iron permease	0.038±0.010	0.119±0.021	0.128±0.020	**0.0121** **0.0081**	**0.0454** **0.0332**
fecR (Fur-, σ^D)	Signal transducer for ferric citrate transport	0.040±0.008	0.026±0.000	0.030±0.004	0.0722 0.1530	0.1600 0.1798
yoeA	Outer membrane receptor for iron compounds	0.044±0.003	0.497±0.135	0.072±0.015	**0.0141** 0.0755	0.0506 0.1165
fhuF (Fur-, σ^D)	Ferric-rhodotorulic acid transporter	0.049±0.007	0.129±0.029	0.060±0.013	**0.0272** 0.2566	0.0801 0.2524
ybiX (Fur-)	Fe(II)-dependent oxygenase superfamily	0.057±0.017	0.083±0.017	0.113±0.017	0.1773 **0.0402**	0.3163 0.0806
entH (Crp, Fur-, σ^D)	Fe siderophore enterobactin synthesis	0.058±0.001	0.034±0.003	0.038±0.009	**0.0005** **0.0447**	**0.0077** 0.0854
entC (Crp, Fur-, σ^D)	Fe siderophore enterobactin synthesis	0.058±0.006	0.125±0.013	0.067±0.015	**0.0042** 0.3036	**0.0243** 0.2853
ybdZ (Fnr, Fur-, Hns, σ^D)	Stimulator of EntF, enterochelin synthesis	0.060±0.014	0.009±0.002	0.037±0.018	**0.0109** 0.1850	**0.0425** 0.2011
feoA (Fnr, Fur-, σ^D)	Ferrous iron transporter	0.060±0.013	0.093±0.017	0.099±0.041	0.0979 0.2114	0.2013 0.2195
fepB (Fur-, RutR-, σ^D)	Iron-enterobactin transporter	0.062±0.004	0.049±0.004	0.080±0.010	**0.0475** 0.0728	0.1190 0.1139
entA (Crp, Fur-, σ^D)	Fe siderophore enterobactin synthesis	0.070±0.006	0.038±0.003	0.062±0.016	**0.0063** 0.3507	**0.0307** 0.3161
entF (Crp, Fur-, σ^D)	Fe siderophore enterobactin synthesis	0.095±0.031	0.029±0.007	0.051±0.016	**0.0548** 0.1394	0.1324 0.1696
entB (Crp, Fur-, σ^D)	Fe siderophore enterobactin synthesis	0.106±0.007	0.032±0.002	0.071±0.028	**0.0003** 0.1440	**0.0068** 0.1734
**Phosphate utilization and uptake**						
gadW (Fnr-, GadX-, Hns-, PhoP, RutR-)	Activates *gadA*, acid resistance, protects in phosphate starvation	0.023±0.002	0.069±0.021	0.119±0.032	**0.0444** **0.0194**	0.1133 **0.0528**
**Motility** [d]						
fliK (CpxRA-, FlhDC, σ^F, σ^D)	Flagellar protein	0.025±0.011	0.009±0.002	0.253±0.038	0.1213 **0.0022**	0.2357 **0.0179**
fliQ (CpxRA-, FlhDC, σ^F, σ^D)	Flagellar protein	0.026±0.006	0.030±0.010	0.128±0.028	0.3788 **0.0125**	0.5676 **0.0413**
fliP (CpxRA-, FlhDC, σ^F, σ^D)	Flagellar protein	0.032±0.003	0.048±0.010	0.279±0.040	0.1055 **0.0017**	0.2127 **0.0163**
fliR (CpxRA-, FlhDC, σ^F, σ^D)	Flagellar protein	0.033±0.004	0.060±0.012	0.044±0.005	**0.0498** **0.0907**	0.1232 0.1297
flhC (CpxRA-, Crp, Fur-, HdfR-, Ihf-, Rcs-)	Activator of flagellar operons	0.033±0.006	0.231±0.020	0.442±0.214	**0.0004** 0.0646	**0.0071** 0.1063
flhE (CpxRA-, FlhDC)	Flagellar protein	0.034±0.005	0.019±0.003	0.386±0.061	**0.0324** **0.0022**	0.0909 **0.0179**
fliJ (CpxRA-, FlhDC, σ^F, σ^D)	Flagellar protein	0.036±0.005	0.013±0.003	0.290±0.005	**0.0095** **0.0000**	**0.0388** **0.0016**
fliM (CpxRA-, FlhDC, σ^F, σ^D)	Flagellar protein	0.042±0.005	0.093±0.004	0.316±0.038	**0.0006** **0.0010**	**0.0086** **0.0139**
flhD (CpxRA-, Crp, Fur-, HdfR-, Ihf-, Rcs-)	Activator of flagellar operons	0.052±0.007	0.667±0.036	0.381±0.198	**0.0000** 0.0858	**0.0028** 0.1253
fliT (CpxRA-, FlhDC, σ^F, σ^D)	Flagellar protein	0.054±0.008	0.040±0.009	0.691±0.091	0.1460 **0.0011**	0.2723 **0.0140**
fliE (CpxRA-, FlhDC, σ^F, σ^D)	Flagellar protein	0.055±0.007	2.266±0.427	0.150±0.089	**0.0033** 0.1757	**0.0211** 0.1972
flgJ (CpxRA-, FlhDC, σ^D)	Flagellar protein	0.063±0.019	0.012±0.003	0.302±0.044	**0.0291** **0.0039**	0.0838 **0.0234**
flgH (CpxRA-, FlhDC, σ^D)	Flagellar protein	0.066±0.020	0.025±0.002	0.582±0.072	0.0567 **0.0012**	0.1357 **0.0143**
fliF (CpxRA-, FlhDC, σ^F, σ^D)	Flagellar protein	0.067±0.005	0.134±0.016	0.361±0.094	**0.0074** **0.0174**	**0.0336** **0.0492**
fliN (CpxRA-, FlhDC, σ^F, σ^D)	Flagellar protein	0.068±0.012	0.124±0.033	0.568±0.043	0.0944 **0.0002**	0.1960 **0.0078**
flgI (CpxRA-, FlhDC, σ^D)	Flagellar protein	0.073±0.019	0.015±0.003	0.392±0.018	**0.0192** **0.0001**	0.0625 **0.0068**
fliI (CpxRA-, FlhDC, σ^F, σ^D)	Flagellar protein	0.073±0.019	0.013±0.002	0.341±0.052	**0.0168** **0.0041**	0.0568 **0.0240**
flhA (CpxRA-, FlhDC)	Flagellar protein	0.104±0.007	0.099±0.006	0.307±0.047	0.3154 **0.0065**	0.4919 **0.0297**
**Other**						
rcsA (GadE, Hns-, RcsB)	Colanic acid capsular biosynthesis activation	0.021±0.002	0.054±0.007	0.106±0.004	**0.0065** **0.0000**	**0.0311** **0.0039**
gapC	Glyceraldehyde 3-phosphate dehydrogenase	0.023±0.006	0.498±0.089	0.163±0.025	**0.0030** **0.0026**	**0.0200** **0.0189**
slp (GadW, GadX, MarA-, σ^D)	Outer membrane lipoprotein	0.030±0.006	0.104±0.011	0.030±0.004	**0.0024** 0.4678	**0.0175** 0.3880
hdeB (GadW, GadX, Hns-, MarA-, σ^D)	Acid-resistance protein	0.031±0.004	0.023±0.000	0.055±0.024	0.0658 0.1876	0.1499 0.2015
gtrB	Prophage bactoprenol glucosyl transferase	0.033±0.010	0.173±0.012	0.395±0.072	**0.0004** **0.0038**	**0.0072** **0.0229**

Table S3. Continued

mtfA	Anti-repressor for DgsA(Mlc)	0.039±0.002	0.119±0.029	0.132±0.029	**0.0256**	**0.0172**	0.0766	**0.0493**
arnA	UDP-L-Ara4N formyltransferase	0.040±0.014	0.032±0.002	0.064±0.020	0.3186	0.1949	0.4958	0.2066
xanP	Xanthine permease	0.040±0.007	0.093±0.016	0.091±0.015	**0.0212**	**0.0195**	0.0671	**0.0529**
yeaP	Putative diguanylate cyclase	0.041±0.021	0.281±0.008	0.099±0.030	**0.0002**	0.0960	**0.0055**	0.1336
fimI (Hns, Ihf, Lrp, σ^D)	Fimbrial protein, type 1 pilus synthesis	0.044±0.012	0.386±0.096	0.127±0.036	**0.0118**	**0.0459**	**0.0447**	0.0865
coaA	Pantothenate kinase	0.044±0.006	0.208±0.026	0.349±0.148	**0.0018**	**0.0538**	**0.0147**	0.0952
dicA	Qin prophage regulator for DicB	0.045±0.010	0.888±0.170	0.565±0.201	**0.0039**	**0.0303**	**0.0231**	0.0682
glnK (GadX, GlnG, σ^N)	Nitrogen assimilation regulatory protein	0.046±0.000	0.187±0.077	0.046±0.000	0.0695	1.0000	0.1552	0.4000
purN (Fnr-, PurR-, σ^D)	Phosphoribosylglycinamide formyltransferase	0.047±0.005	0.341±0.022	0.183±0.021	**0.0001**	**0.0016**	**0.0041**	**0.0157**
spr	Outer membrane lipoprotein	0.049±0.010	0.927±0.031	0.341±0.116	**0.0000**	**0.0332**	**0.0014**	0.0721

[a] Bacteria were treated with albumin (100 µg/ml, control), PGRP (PGLYRP4, 100 µg/ml), or gentamicin (5 µg/ml), for 30 min or CCCP (800 µM) for 15 min at 37°C, and gene expression was determined by whole genome expression arrays. Genes are listed from the most to the least down-regulated in each group. The numbers are mean ratios ± SEM of the gene expression signals in PGRP-, gentamicin-, or CCCP-treated bacteria to control albumin-treated bacteria, obtained from 3 independent whole genome expression arrays experiments. All genes in PGRP-treated bacteria were expressed significantly lower than in control bacteria at $P<0.001$ by two sample one-tailed *t*-test (not shown), and all of these genes were expressed significantly lower than in control at FDR $q\leq 0.03$ (not shown). The entire whole genome expression array data have been deposited in NCBI GEO under the accession number GSE44211.

[b] *P* values (two sample one-tailed *t*-test) for the differences in gene expression in PGRP-treated bacteria *versus* gentamicin- or CCCP-treated bacteria (numbers on the left or on the right, respectively) are shown as indicated, with $P\leq 0.05$ in bold.

[c] FDR (false discovery rate) *q* values for the differences in gene expression in PGRP-treated bacteria *versus* gentamicin- or CCCP-treated bacteria (numbers on the left or on the right, respectively) are shown as indicated, with $q\leq 0.05$ in bold.

[d] FlhDC and FlhDC-controlled motility genes are negatively regulated by CpxRA, but they do not contain the CpxR-binding site.
