# Supplementary material for: Peptidoglycan Recognition Proteins Kill Bacteria by Inducing Oxidative, Thiol, and Metal Stress
Source: PLoS Pathog. 2014 Jul 17;10(7):e1004280. doi: 10.1371/journal.ppat.1004280 (PMC4102600; doi:10.1371/journal.ppat.1004280)
Supplement: Table S4 — Top B. subtilis genes down-regulated by PGRP, gentamicin, and CCCP. (PDF) [file ppat.1004280.s011.pdf]

Table S4. Top *B. subtilis* genes down-regulated by PGRP, gentamicin, and CCCP [a].

Gene (regulators)	Function	PGRP	Gentamicin	CCCP	*P* (*t*-test) [b]		FDR *q* [c]	
**Phosphate utilization and uptake**								
tuaA (PhoPR, σ^A)	Teichuronic acid synthesis	0.007±0.000	0.007±0.000	0.007±0.000	1.0000	0.1870	0.2400	0.1278
tuaC (PhoPR, σ^A)	Teichuronic acid synthesis	0.008±0.001	0.007±0.000	0.011±0.004	0.0697	0.2791	0.0859	0.1602
pstA (PhoPR, σ^A)	High-affinity ABC phosphate uptake	0.011±0.001	0.008±0.000	0.023±0.013	**0.0315**	0.1899	0.0632	0.1287
pstC (PhoPR, σ^A)	High-affinity ABC phosphate uptake	0.012±0.004	0.008±0.000	0.014±0.004	0.1712	0.4032	0.1273	0.1984
tuaD (PhoPR, σ^A)	Teichuronic acid synthesis	0.013±0.004	0.009±0.003	0.013±0.005	0.2361	0.4670	0.1513	0.2166
tuaH (PhoPR, σ^A)	Teichuronic acid synthesis	0.013±0.002	0.014±0.004	0.016±0.004	0.3897	0.2601	0.2101	0.1544
tuaB (PhoPR, σ^A)	Teichuronic acid synthesis	0.014±0.003	0.009±0.000	0.009±0.001	0.0804	0.1244	0.0911	0.1075
phoB (PhoPR, σ^E)	Alkaline phosphatase (phosphate acquisition)	0.018±0.000	0.021±0.003	0.018±0.000	0.1870	1.0000	0.1319	0.2250
tuaE (PhoPR, σ^A)	Teichuronic acid synthesis	0.018±0.001	0.019±0.002	0.016±0.002	0.4084	0.2734	0.2162	0.1586
tuaG (PhoPR, σ^A)	Teichuronic acid synthesis	0.020±0.004	0.019±0.003	0.021±0.006	0.3982	0.4414	0.2129	0.2094
pstS (PhoPR, σ^A)	High-affinity ABC phosphate uptake	0.021±0.010	0.006±0.002	0.057±0.030	0.1250	0.1588	0.1080	0.1205
pstBB (PhoPR, σ^A)	High-affinity ABC phosphate uptake	0.022±0.009	0.011±0.002	0.071±0.049	0.1645	0.1895	0.1246	0.1287
pstBA (PhoPR, σ^A)	High-affinity ABC phosphate uptake	0.027±0.009	0.018±0.000	0.060±0.037	0.1870	0.2227	0.1319	0.1401
tuaF (PhoPR, σ^A)	Teichuronic acid synthesis	0.035±0.007	0.022±0.002	0.029±0.004	0.0851	0.2603	0.0933	0.1534
**Motility**								
motA (σ^D)	Flagellar motor rotation	0.027±0.000	0.746±0.097	0.690±0.113	**0.0009**	**0.0021**	**0.0148**	**0.0219**
flhO (σ^D)	Fagellar protein	0.051±0.015	1.150±0.229	0.837±0.022	**0.0044**	**0.0000**	**0.0262**	**0.0032**
yvyC (σ^D)	Fagellar protein	0.066±0.000	0.316±0.048	0.675±0.101	**0.0032**	**0.0019**	**0.0227**	**0.0210**
flhB (σ^D)	Fagellar protein	0.092±0.025	0.363±0.088	0.617±0.152	**0.0206**	**0.0137**	**0.0524**	**0.0470**
flhA (σ^D)	Fagellar protein	0.118±0.020	0.321±0.093	0.360±0.085	**0.0497**	**0.0251**	0.0768	0.0577
fliR (σ^D)	Fagellar protein	0.132±0.038	0.399±0.071	0.568±0.081	**0.0151**	**0.0042**	**0.0450**	**0.0297**
motB (σ^D)	Flagellar motor rotation	0.133±0.014	1.157±0.253	0.631±0.306	**0.0078**	0.0893	**0.0334**	0.0936
fliD (σ^D)	Fagellar protein	0.138±0.048	1.427±0.393	0.832±0.258	**0.0156**	**0.0285**	**0.0459**	0.0600
flgB (CodY, Spo0A, σ^A, σ^D)	Flagellar basal-body rod protein	0.140±0.005	0.824±0.210	0.755±0.034	**0.0156**	**0.0000**	**0.0459**	**0.0048**
fliZ (σ^D)	Fagellar protein	0.146±0.034	0.391±0.040	0.638±0.156	**0.0046**	**0.0183**	**0.0266**	**0.0514**
yvyG (σ^D)	Fagellar protein	0.157±0.027	5.050±1.966	1.108±0.374	**0.0338**	**0.0320**	0.0651	0.0628
fliY (σ^D)	Fagellar protein	0.167±0.029	0.383±0.071	0.784±0.193	**0.0237**	**0.0171**	0.0564	**0.0507**
flhP (σ_D)	Fagellar protein	0.184±0.046	1.572±0.166	0.469±0.042	**0.0006**	**0.0050**	**0.0124**	**0.0311**
fliG (σ^D)	Fagellar protein	0.193±0.082	0.622±0.169	0.563±0.136	**0.0424**	**0.0397**	0.0706	0.0685
flhF (σ^D)	Fagellar protein	0.193±0.017	0.398±0.099	0.497±0.174	**0.0550**	0.0788	0.0798	0.0893
yvyF (σ^D)	Fagellar protein	0.195±0.047	1.816±0.401	1.378±0.184	**0.0080**	**0.0017**	**0.0336**	**0.0199**
**Iron uptake**								
yxeB (Fur-)	Hydroxamate siderophore ABC Fe uptake	0.060±0.012	0.036±0.000	0.615±0.120	0.0595	**0.0051**	0.0813	**0.0312**
dhbB (Fur-, AbrB, σ^A)	Siderophore bacillibactin synthesis	0.069±0.018	0.033±0.000	0.095±0.062	0.0602	0.3529	0.0813	0.1834
dhbF (Fur-, AbrB, σ^A)	Siderophore bacillibactin synthesis	0.076±0.030	0.021±0.000	0.062±0.041	0.0682	0.3947	0.0856	0.1932
besA (Fur-, AbrB, σ^A)	Trilactone hydrolase, Fe acquisition	0.080±0.036	0.013±0.000	0.067±0.022	0.0682	0.3824	0.0865	0.1922
dhbC (Fur-, AbrB, σ^A)	Siderophore bacillibactin synthesis	0.083±0.029	0.013±0.000	0.062±0.049	**0.0364**	0.3659	0.0666	0.1872
ykuP (Fur-, σ^A)	Flavodoxin, induced by iron starvation	0.084±0.037	0.016±0.000	0.034±0.017	0.0717	0.1451	0.0867	0.1158
yfiY (Fur-)	ABC transporter for schizokinen and arthrobactin	0.086±0.015	0.062±0.010	0.236±0.107	0.1203	0.1195	0.1061	0.1065
dhbE (Fur-, AbrB, σ^A)	Siderophore bacillibactin synthesis	0.099±0.036	0.039±0.000	0.098±0.059	0.0823	0.4946	0.0921	0.2243
efeU (Fur-)	Ferrous ion permease	0.104±0.032	0.057±0.000	0.204±0.054	0.1073	0.0934	0.1019	0.0944
dhbA (Fur-, AbrB, σ^A)	Siderophore bacillibactin synthesis	0.117±0.056	0.016±0.000	0.048±0.028	0.0730	0.1667	0.0874	0.1234
yhfQ (Fur-)	Iron/citrate ABC transporter, Fe uptake	0.120±0.061	0.048±0.002	0.321±0.025	0.1506	**0.0190**	0.1186	**0.0525**

Table S4. Continued

fhuD (Fur-)	Ferrichrome ABC transporter	0.128±0.027	0.054±0.000	0.667±0.071	**0.0264**	**0.0010**	0.0588	**0.0163**
efeO (Fur-)	Lipoprotein binding ferrous or ferric iron for uptake	0.133±0.033	0.093±0.011	0.151±0.066	0.1580	0.4074	0.1220	0.1994
fhuC (Fur-)	Ferrichrome ABC transporter	0.137±0.025	0.117±0.006	0.151±0.039	0.2434	0.3900	0.1545	0.1946
**Other**								
artP	Arginine ABC transporter	0.034±0.004	0.066±0.007	0.074±0.016	**0.0094**	**0.0092**	**0.0350**	0.0671
yxkC (TnrA, σ^D)	Unknown	0.050±0.000	0.495±0.075	1.126±0.246	**0.0020**	**0.0059**	**0.0191**	**0.0328**
yolA (AbrB)	Unknown	0.061±0.000	0.592±0.119	1.174±0.289	**0.0055**	**0.0092**	**0.0290**	**0.0393**
rapF (CcpA, ComA)	Aspartate phosphatase response regulator	0.062±0.007	0.199±0.043	0.463±0.150	**0.0169**	**0.0280**	**0.0476**	0.0596
cwlQ (σ^D)	Muramidase/transglycosylase, cell wall synthesis	0.065±0.015	0.923±0.117	0.594±0.115	**0.0010**	**0.0052**	**0.0149**	**0.0317**
yukL	Protein similar to antibiotic synthetase	0.069±0.034	0.018±0.000	0.05±0.037	0.1005	0.3903	0.0991	0.1946

[a] Bacteria were treated with albumin (100 µg/ml, control), PGRP (PGLYRP4, 100 µg/ml), or gentamicin (5 µg/ml), for 30 min or CCCP (800 µM) for 15 min at 37°C, and gene expression was determined by whole genome expression arrays. Genes are listed from the most to the least down-regulated in each group. The numbers are mean ratios ± SEM of the gene expression signals in PGRP-, gentamicin-, or CCCP-treated bacteria to control albumin-treated bacteria, obtained from 3 independent whole genome expression arrays experiments. All genes in PGRP-treated bacteria were expressed significantly lower than in control bacteria at P<0.001 by two sample one-tailed t-test (not shown), and 90% of these genes were expressed significantly lower than in control at FDR $q \le 0.05$ (not shown). The entire whole genome expression array data have been deposited in NCBI GEO under the accession number GSE44212.

[b] P values (two sample one-tailed t-test) for the differences in gene expression in PGRP-treated bacteria *versus* gentamicin- or CCCP-treated bacteria (numbers on the left or on the right, respectively) are shown as indicated, with $P \le 0.05$ in bold.

[c] FDR (false discovery rate) q values for the differences in gene expression in PGRP-treated bacteria *versus* gentamicin- or CCCP-treated bacteria (numbers on the left or on the right, respectively) are shown as indicated, with $q \le 0.05$ in bold.
