# Supplementary material for: Peptidoglycan Recognition Proteins Kill Bacteria by Inducing Oxidative, Thiol, and Metal Stress
Source: PLoS Pathog. 2014 Jul 17;10(7):e1004280. doi: 10.1371/journal.ppat.1004280 (PMC4102600; doi:10.1371/journal.ppat.1004280)
Supplement: Table S5 — Bacterial strains used in this study. (PDF) [file ppat.1004280.s012.pdf]

**Table S5. Bacterial strains used in this study.**

| Strain | Relevant genotype | Source or reference |
|---|---|---|
| ***E. coli*** | | |
| MG1655 | Wild type parental K-12 strain (ATCC 700926) for mutants | ATCC |
| W3110 | Wild type parental K-12 strain for mutants | 28, 77[a] |
| hpx$^{-}$ (Δ*katG*Δ*katE*Δ*ahpCF,* LC106) | Δ*(katG17::Tn10)1 (ahpC-ahpF')del kan::'ahpF* Δ*(katE12::Tn10)1* | 15 |
| Δ*copA*Δ*cueO*Δ*cusCFBA* (GR17) | Δ*copA*::Km Δ*cueO*Δ *cusCFBA*::Cm F- λ-IN(rrnD-rrnE)1 rph-1 | 77 |
| Δ*gshA* (KCI1220) | Δ*gshA20::*Km | 30 |
| Δ*recA* (Lem17) | *recA56 srl300::Tn10tet* | 30 |
| Δ*zntA*Δ*zitB* (GG48) | Δ*zitB*::Cm Δ*zntA*::Km | 28 |
| NDM-1 (BAA-2452) | Resistant to all β-lactams and aminoglycosides | ATCC |
| ***B. subtilis*** | | |
| 168 | Wild type | 7 |
| CU1065 | Wild type parental strain for mutants | 23 |
| hpx$^{-}$ (Δ*katA*Δ*ahpCF*) | Δ*katA::*TC Δ*ahpCF:*Km | This study |
| Δ*bshC* (HB11212) | Δ*bshC::mls* | This study |
| Δ*cadA* (HB11393) | Δ*cadA*::Km | This study |
| Δ*copZA* (HB5002) | Δ*copZA*::Km | 29 |
| Δ*czcD* (HB11394) | Δ*czcD*::Tet | This study |
| Δ*czcD*Δ*cadA* (HB11395) | Δ*czcD*::Tet Δ*cadA*::Km | This study |
| ***S. aureus*** | | |
| Newman D2C | Wild type strain (NCTC 10833) | ATCC |
| MRSA (NARSA NRS384) | Resistant to penicillins, cephalosporins, macrolides, and aminoglycosides | NARSA |

[a] 77. Grass G, Rensing C (2001) Genes involved in copper homeostasis in *Escherichia coli*. J Bacteriol 183: 2145-2147.
