# Supplementary material for: Peptidoglycan Recognition Proteins Kill Bacteria by Inducing Oxidative, Thiol, and Metal Stress
Source: PLoS Pathog. 2014 Jul 17;10(7):e1004280. doi: 10.1371/journal.ppat.1004280 (PMC4102600; doi:10.1371/journal.ppat.1004280)
Supplement: Table S6 — E. coli and B. subtilis primers for qRT-PCR. (PDF) [file ppat.1004280.s013.pdf]

Table S6. *E. coli* and *B. subtilis* primers for qRT-PCR.

Gene	Forward primer (5'–3')	Reverse primer (5'–3')
E. coli		
ahpF	AGTTCACCTCGCTGGTACTGG	TCTGGAAGGTGCCGCCGTCAAT
arsB	GCCTGCCGCTTATTGTCTCCA	TCCCACCAGCAGAAGCAGTAA
arsC	ATACGCTGGAGATGATCCGCA	CCAGCGGCGTCACCACAATCG
arsR	TTCTTGCTGATGAAACCCGTCTGG	CAATAGCCCGCTTTCACGCAG
clpB	AGCGATTGAACAAATGCGTGGAGG	CAGACCTTCAACGATGGCAGT
copA	TGCTGAACGGTGCGGCGACGAT	TTCGTCAGTAACCAGGCGTGC
cueO	ATCAACCTGCCGCTACCTGCT	CAGCGTGTTGCGGGTAGATTG
degP	GCTCTCCGTTCTGCCAGGGTG	GTCAGGTTTTTCGGGTTCTGG
fumC	ATTCCCGCTGGCTATCTGGCA	CTCATTCAGTGTCTGTGTCAGG
grxA	TTGCCCTTACTGTGTGCGTGC	GCAGCAAAATCGGTATAGCC
hspQ	AGGTTATCTCGGAGTGGTCGT	GGAGTTGTTTGCGGATGGTCT
htpG	GGCTGGCGAAGGTGAATACAC	ACTTGTTACGAGTCCACAGC
htpX	TGGCATTACGATCTGTTGGCGG	TTACCGCCTCGGCTTCATCCG
ibpB	CCGCCGTACAACATTGAGAAAAGC	GCTGCGATGGGTTCAGGCTCA
iscU	GGGTTCCTTTGACAACAACGAC	GGCGGCAGTTCAAGTTCTTCA
marA	AAGTGTCAGAGCGTTCGGGTT	ATGCGGCGGAACATCAAAGT
marB	CACTTTCATCCGCAATAGCAGC	CATCCGACTTATCACTGCCAG
metN	AACCGAGGGTAGCGTGCTGGT	CCAAGACCAACCAATGACAG
mmuP	GCTTTACCGCCGCTGGATTCT	GTTCACTGCCACCATAGTCATC
ndh	GACTGGCTCGCTTGATGAAGG	ATTCAGCATCTCCTGGTGGAAGC
oxyS	CCTGGAGATCCGCAAAAGTTCA	AACGGAGCGGCACCTCTTTTA
rcnA	CTCAATCAGCAGAACCGTGGC	CTCTCTACCATCAAAGCGTCGT
ybdL	GTAACGGCAGGGGCGACGGAA	TGGGGTTATGCGGAGTGTTGA
yhbW	GTCGGTATTGATCGGCTATCTG	AACCAGTCCACCAGCTCCGCC
yhcN	GCACAAAATCGTGAAGCAATCGGG	TACAGTTCAGCCGTAGCGTGC
B. subtilis		
ahpF	GGAGAAGACACTGGCGTGACTT	TCGGGTTCAGCACGCTCATCAT
arsB	GCGGCAGGATTAGTTGCTTTC	GGACAACATCTAACGGCAAGC
arsC	AAATGGCTGAAGGATGGGCT	CATCAAAACCCCAATGCTCACG
cadA	GCTCGGTTTCTTCGGTGGAAT	ACGCACAGATTCAGCAATCG
copA	GACACCAGTCCAGTTTCTGAT	GATTTGCTCCTGTCCGTCTCT
cypE	GGAGGATACTGTTCTAGGCGG	CAAGACCGAGAACCATTGTC
czcD	GGATTATGATGAGTGGCGGAGA	GTCAACAACTGCGTGGCAAGAA
hisF	GCTTGCCATTCCGTTTACAGTC	TTTCTGCCGCCGTGCGTGTAGA
hisH	TATGGTCTCAGAGGGAAGGCT	GGAATGAACGAAGTAAGCGTAGCC
hisI	GAACACGCAAGCCGTCAAAGGT	CGGAAGAGGTACGTGGTGTAAG
htrA	CACCAGCGATTGTCGGTATTAC	ACCCTGAACCGCTTTCTGTATC
htrB	GCTGACTGTTACGCTATACAACGGAG	CTGTTGATGCCGATGACCTGG
katA	TTTCACCAGAGTCACTGCACC	GTGACATCAAACGGATCGAAAC
katE	ATGGTGATGTGGACGATGTCTG	ACGGCGGTGGAAATCTGGAT
ldh	GCGGCACAACACTTGATTCTG	CTCATCGCAACCCCATAATAAGTCGC
nfrA	GGTTAGCTTGATTGATGCGGC	CCTGCTTTGGCAATCTCGGTT
nhaX	TGACAGCCATTGATCTTGC	ATCAGCGGATCAGGAACACTTG
ybcD	CATCGTAGCTGATGACGCTGAC	CTATCGGTCCCATCCAGACATC
ybcF	GGTGCCAGTATTTCACATCC	GCCATTCATCGACCGTACCACCT
yqjM	AGGACGAATCACTGACCAAGAC	GCTTGAACTCCTGGACCGTTTC
yraA	TGGCTACAGTGTTGTGGCTATC	GACGGTCATCTGCACGAAGCAAAT
ytrB	GACGCAATGGATCGGGCAAAA	GGTCTCGCCGCAAACGACAGAA
ytrC	GGCGGTTTTGTGATTGTGCTGTCA	AATAACTCTCAGGGGAAGGG
ytrD	GGGAGTCGTTTTGGCGGTCAG	CAGGCAGGCTGATGAGTAAAAACG
ytrE	ATTGTCGGACGAAGCGGCTCA	GAGCGGCGTGGTTTTCCAGA
ytrF	GGCGGAAATGTTGAAACGAAC	TCACCGCCATCGTCATCGTGTT
ywcH	CGTGTTGGATCAGGAGGAGTCA	GCAGCGGTCAGATTCGGAAAAC
yxnA	GACAGGGGAACGGTCATTCAAT	CGGATAGATCATGGAGCGGTAA
